# Precision fMRI reveals that the language network exhibits adult-like left-hemispheric lateralization by 4 years of age

**DOI:** 10.1101/2024.05.15.594172

**Published:** 2024-05-15

**Authors:** Ola Ozernov-Palchik, Amanda M. O’Brien, Elizabeth Jiachen Lee, Hilary Richardson, Rachel Romeo, Benjamin Lipkin, Hannah Small, Jimmy Capella, Alfonso Nieto-Castañón, Rebecca Saxe, John D. E. Gabrieli, Evelina Fedorenko

**Affiliations:** 1 McGovern Institute for Brain Research, MIT, Cambridge, MA 02139, United States; 2 Program in Speech and Hearing Bioscience and Technology, Harvard University, Cambridge, MA 02138, United States; 3 Department of Brain and Cognitive Sciences, MIT, Cambridge, MA 02139, United States; 4 School of Philosophy, Psychology, and Language Sciences, University of Edinburgh, Edinburgh, EH8 9JZ, United Kingdom; 5 Department of Human Development and Quantitative Methodology, University of Maryland, College Park, MD 20742, United States; 6 Department of Cognitive Science, Johns Hopkins University, Baltimore, MD, 21218, United States; 7 Department of Psychology and Neuroscience, University of North Carolina at Chapel Hill, Chapel Hill, NC 27599, United States

## Abstract

Left hemisphere damage in adulthood often leads to linguistic deficits, but many cases of early damage leave linguistic processing preserved, and a functional language system can develop in the right hemisphere. To explain this early apparent equipotentiality of the two hemispheres for language, some have proposed that the language system is bilateral during early development and only becomes left-lateralized with age. We examined language lateralization using functional magnetic resonance imaging with two large pediatric cohorts (total n=273 children ages 4-16; n=107 adults). Strong, adult-level left-hemispheric lateralization (in activation volume and response magnitude) was evident by age 4. Thus, although the right hemisphere can take over language function in some cases of early brain damage, and although some features of the language system do show protracted development (magnitude of language response and strength of inter-regional correlations in the language network), the left-hemisphere bias for language is robustly present by 4 years of age.

In a truly incredible feat, approximately six months after they are born, human babies begin to recognize words for common objects^1^, and toward their first birthday, they utter their first words^2^. Over the following couple of years, their vocabularies explode and they learn to combine words into phrases and sentences. By about 4 years of age, typically developing children can understand and express complex ideas through language^3,4^. But is the brain infrastructure for language the same in these young but already quite competent language users as in adults?

In adult brains, language processing, including both comprehension and production, draws on a specialized network of frontal and temporal areas^5–7^. In the vast majority of individuals, this network is lateralized to the left hemisphere, which manifests as a) stronger and more spatially extensive responses to language in the left hemisphere (see^8^ for data from >800 individuals), and b) a greater likelihood of linguistic deficits (aphasia) following left hemisphere damage in adulthood^9–11^. One important controversy about the development of language-processing mechanisms concerns the degree of left-hemispheric lateralization of the language network in children.

According to one influential proposal, the language system starts out as bilateral and only becomes left-lateralized with age^12,13^. This proposal is motivated by the apparent equipotentiality of the two hemispheres for language early in life: damage to the left hemisphere in childhood can leave linguistic functions preserved, with the right hemisphere homotopic areas taking over^14–17^ (cf.^18,19^). This proposal also makes predictions about language development in typical brains, which can be evaluated using functional brain imaging. In particular, the fronto-temporal networks in the two hemispheres should be similar in their responses to language during early development. Indeed, some neuroimaging studies that have examined responses to language from about age 4 years onwards have reported more bilateral responses at younger ages and more strongly left-lateralized responses with age^20–27^. However, this empirical claim is controversial: other studies have reported already left-lateralized responses to language in children^28–30^, by four years of age^31^.

The complexity of the empirical landscape may have to do with i) the predominant reliance of past fMRI studies on the traditional group-averaging approach, which suffers from low sensitivity, functional resolution, and interpretability^32–34^, and ii) the diversity of experimental paradigms, which makes comparisons across studies challenging. Some paradigms further conflate language processing with lower-level speech perception or with general task demands, both of which draw on bilateral systems even in adults (speech perception^35^; task demands^36^). For example, many language experiments involve not only comprehension of language but also meta-linguistic judgments or comprehension questions, which are known to recruit the domain-general Multiple Demand network^37^. If these meta-linguistic tasks are more difficult for children compared to adults, they may recruit the Multiple Demand network to a greater degree, which would lead to the appearance of more bilateral responses in children (we return to this issue in the Discussion).

Here, we characterize the development of the language network across two independent pediatric cohorts (Dataset 1: 206 children, aged 4-14, and 91 adults; Dataset 2: 67 children, aged 4-16, and 16 adults). We use a robust individual-subject fMRI approach (‘precision fMRI’^32,34,38,39^) and an extensively validated language ‘localizer’ paradigm^8,32,40^, which robustly isolates the language areas from both lower-level speech areas and domain-general areas sensitive to task demands^41,42^. Although our main focus is on lateralization, we also examine other properties of the language network, for which the developmental trajectory remains debated in the developmental neuroscience literature. These include the language network’s topography (e.g., how early does the frontal component of the language network emerge?^23,25,43,44^), its selectivity for language, and the degree of functional connectivity among the different language regions^23,45^.

To foreshadow the results, across both datasets, we found that left-hemisphere lateralization based on (1) response magnitude and (2) volume of activation was already adult-like by four years of age (see SI-10 for generalization to a third, previously published dataset^26^). In contrast, other aspects of the brain organization for language show a clear developmental trajectory: both the magnitude of response to language and the strength of inter-regional correlations increase from early to middle to late childhood, at which point they reach adult levels. These findings suggest that the capacity of the right-hemisphere frontal and temporal areas to support language processing in cases of early left-hemisphere damage is not due to the language system being typically more bilateral in young children. More generally, these results establish the normative developmental trajectory for key individual-level neural markers of language processing, thus laying the groundwork for future investigations of linguistic processing at earlier ages and in developmental populations with language disorders.

## Results

**Figure 1.**
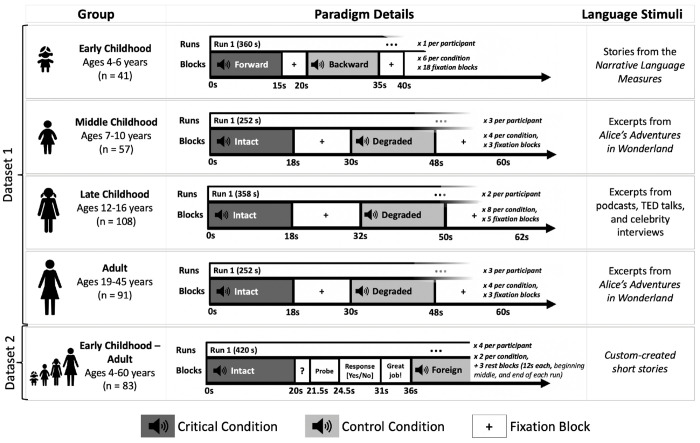
A summary of the design and procedure for the language localizer variants used for participants in Datasets 1 and 2 (see Methods-Section2 and SI-1 for details). In Dataset 1, the paradigm was identical between the Middle Childhood and Adult groups. In Dataset 2, the paradigm was identical across age groups. Importantly, the language localizer contrasts have been previously established to be robust to variation in the materials, task, modality of presentation, specific language, and the nature of the control condition^8,32,40,49^, which means that data from these experiments can be straightforwardly combined and compared.

All participants performed an extensively validated language localizer task^32^ based on a contrast between listening to short age-appropriate stories/passages (the critical condition) and a perceptually similar condition where linguistic content is not comprehensible (the control condition) (Figure 1; see Methods-Section2 and SI-1 for details). This localizer has been shown to selectively engage language areas, and not to engage other nearby functional areas, such as the lower-level speech perception areas^35,46^ and areas of the domain-general Multiple Demand network^47,48^. All statistical analyses were performed on the neural measures of language processing extracted from individual activation maps from the language localizer (see Methods-Section7), including three measures of language lateralization: inter-hemispheric difference in the magnitude of neural (BOLD signal) response, in the volume of activation, and, for completeness, in the strength of inter-regional functional correlations during naturalistic cognition (resting state).

### 1. By age 4, the left-hemisphere language network shows adult-like functional topography.

**Figure 2.**
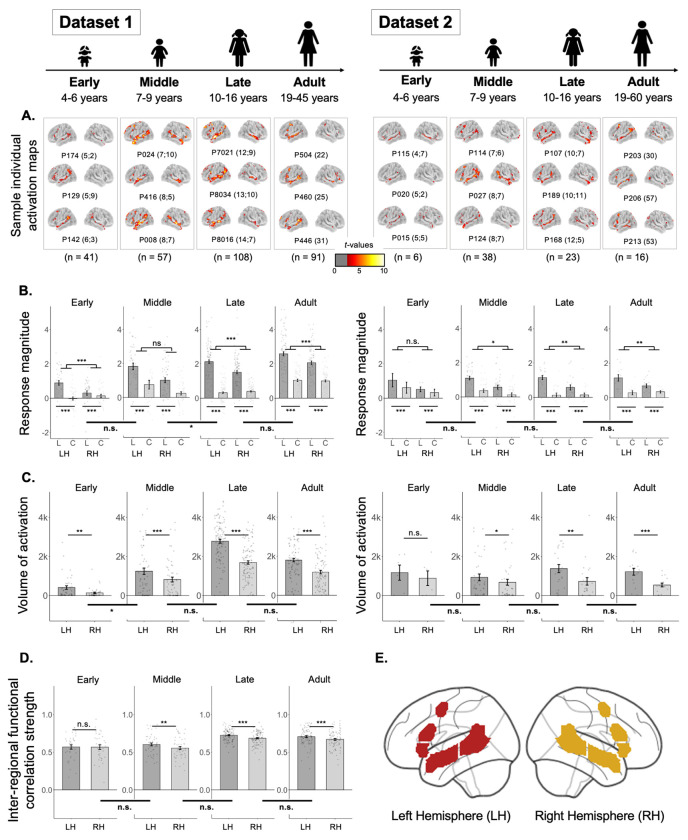
Responses to language and the strength of inter-regional functional correlations in the left hemisphere (LH) and right hemisphere (RH) language network in each age group. **A. Sample individual whole-brain activation maps** for the Language>Control contrast for participants in Datasets 1 and 2 (two broad columns) in each age group (four columns for each dataset). All maps are thresholded at the uncorrected whole-brain level of *p* < 0.01. For each age group in each dataset, we show three sample participants (all individual activation maps are available at https://osf.io/3mvpx/). Participants’ ages are shown in parentheses next to the participants’ unique identifiers (e.g., the age of participant P174 for the Early Childhood group in Dataset 1 is 5 years and 2 months). (These maps are used for visualization only; all statistical analyses are performed on neural measures extracted from these maps, as described in Methods-8.) **B. Magnitude of response (in % BOLD signal change) to the Language condition (“L”, dark grey) and the Control condition (“C”, light grey)** relative to the fixation baseline (zero) in the LH and RH language network in each age group in Datasets 1 and 2. Significant Language>Control effects are marked for each hemisphere in each age group with asterisks below the x-axis (Table 1). Here and in C-D, significant effects of hemisphere are marked for each age group with asterisks above the bars in each plot (Table 2); and significant differences in the effects of hemisphere between age groups are marked on horizontal thick lines below the x-axis that straddle adjacent pairs of bar graphs (Table 3). **C. Volume of activation (number of significant voxels; threshold: p<0.01) for the Language>Control contrast** in each hemisphere in each age group in Datasets 1 and 2. **D. Strength of inter-regional functional correlation (Pearson’s moment correlation)** among the language regions in each hemisphere in each age group in Dataset 1 (no resting state data were collected for Dataset 2). **E.** For all bar graphs (across B-D), the parcels that were used to define individual fROIs (B, D) or to constrain the voxel counts (C) are shown in dark red (LH parcels) and gold (RH parcels) (see Methods-6 for details); for all bars, dots correspond to individual participants, and error bars indicate standard errors of the mean by participant. Significance: ***=*p*<0.001; **=*p*<0.01; *=*p*<0.05 (see Tables 1, 2 and 3 for details).

Because of past claims that the frontal component of the language network exhibits protracted development^44,50^, we first examined the general topography of the language responses across our different age groups, to decide which component(s) to focus on for the critical questions about language lateralization. As shown in Table 1 and Figure 2A, reliable responses to language processing—in both temporal and frontal components—are robustly present in children, including in the youngest pediatric group examined here (4-6 year-olds).

In particular, consistent with what has been reported previously in adults (e.g., see^40^ for the results for the adult group in Dataset 1), participants in each pediatric group showed a reliably stronger response during the Language condition compared to the Control condition (Figure 2B). This effect held across the LH language network as a whole in both datasets and every age group (all *p*s<0.001; Table 1). The effect also held for the temporal and frontal components of the network separately, again in both datasets and every age group (all *p*s<0.001, except the early group in Dataset 2 (n=6): *p*s<0.01; Table 1). (For the parallel analyses of the homotopic RH language network, see SI-2.)

These results suggest that we can examine both the frontal and temporal components of the language network for our critical question of language lateralization across childhood, instead of restricting our analyses to the temporal component.

### 2. The language network exhibits an adult-like left-hemispheric bias by age 4.

In adults, the left-hemispheric (LH) bias for language manifests as a stronger (higher magnitude) and more spatially extensive (greater volume of significant voxels) response to language in the LH language areas. We examined both of these measures for each age group across development.

The effect of hemisphere on the magnitude of the Language>Control effect held in both datasets and all age groups (*p*s<0.05; Table 2A, Figure 2B), except the middle childhood group in Dataset 1 and the early childhood group in Dataset 2. The effect of hemisphere on the volume of activation for the Language>Control contrast also held in both datasets and in all age groups (*p*s<0.05; Table 2B, Figure 2C), except the early childhood group in Dataset 2. The lack of significant effects in the early childhood group in Dataset 2 is likely due to a small sample size of n=6 (note though that the mean inter-hemispheric differences are similar in size to other groups; e.g., in the middle childhood group, there are, on average, 32% more significant Language>Control voxels in the LH than the RH, and in the early childhood group, there are 37% more voxels in the LH than the RH). For the groups that showed lateralized responses at the network level, the effects also held for the temporal and frontal components of the network separately, for both the magnitude of the Language>Control effect and the volume of activation effect (all *p*s<0.001; SI-3).

In addition to exhibiting left-lateralized responses during language tasks, adults also manifest a left-hemispheric bias in the strength of inter-regional correlations within the language network during naturalistic cognition^40,47,48,51^. In Dataset 1, which had naturalistic (resting state) data, the LH regions were more robustly inter-correlated than the RH regions in all groups (*p*s<0.01; Table 2C, Figure 2D), except the early childhood group (see SI-4 for evidence that participants in all pediatric groups showed reliable inter-regional correlations for both local, within-lobe pairs and distant, frontal-to-temporal, pairs: all *p*s<0.001). So, the degree of lateralization as measured by inter-regional functional correlations is the only measure that shows a potential developmental change, with a hemispheric bias not emerging until middle childhood (but see next section and Discussion).

**Figure 3.**
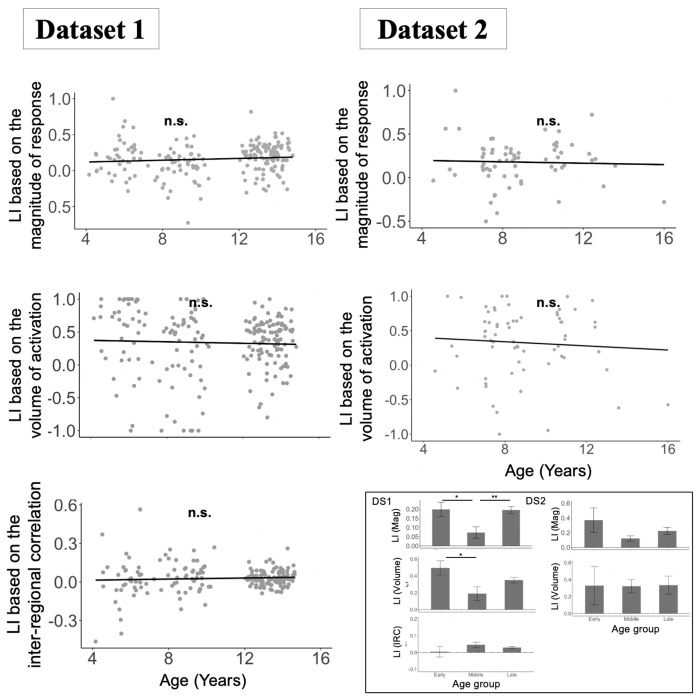
Stability of language network lateralization indices (LI) across age. For each of the three measures, the LI was calculated by using the formula (LH − RH) / (LH + RH), where LH and RH represent left and right hemisphere language network and then averaging LI across fROIs for each participant. For each measure of LI (three rows; magnitude of response, volume of activation, inter-regional correlation), across the two datasets (two broad columns; Dataset 1 (DS1) and Dataset 2 (DS2)), we fit a linear regression model predicting the LI from age as a continuous variable. In addition to the continuous analyses, we also fit linear regression models using age group as a categorical predictor, again including random intercepts for participants and fROIs, followed by inter-group pairwise comparisons adjusted using Tukey’s method. For the magnitude of response measure, we used the magnitude of the top 10% of tvalues from the Language>Control contrast. The inset bar plots show the mean LI and standard error of the mean by group. Significant effects are marked with asterisks (* = *p*<0.05; ** = *p*<0.01; ns = not significant; see Table 3 for details).

To test for age-related changes in lateralization, we used measures that control for overall group differences in the magnitude of effects, which vary with age (Figure 2B–D; Section 3). A standard way to control for these differences is to use laterality index (LI) measures, which are calculated by dividing the difference (in some effect of interest) between the left and right hemispheres by the sum of the effects in the left and right hemispheres^22,52,53^. As shown in Figure 3 and Table 3, we find no significant association between age and degree of lateralization in any of the three measures (magnitude and volume of activation for the Language>Control contrast or strength of inter-regional correlations for resting state data) when treating age as a continuous variable. Similarly, in pairwise group comparisons between children (in different age groups) and adults, we did not find evidence of significant differences in any lateralization measures, across datasets. The few significant group differences that we observed among the childhood groups did not follow the predicted age trajectory (e.g., the middle childhood group in Dataset 1 showed slightly lower lateralization compared to both the early childhood group and the late childhood group).

### 3. Response magnitude and inter-regional correlations increase between ages 4 and 16 and reach maturity by late childhood.

**Figure 4.**
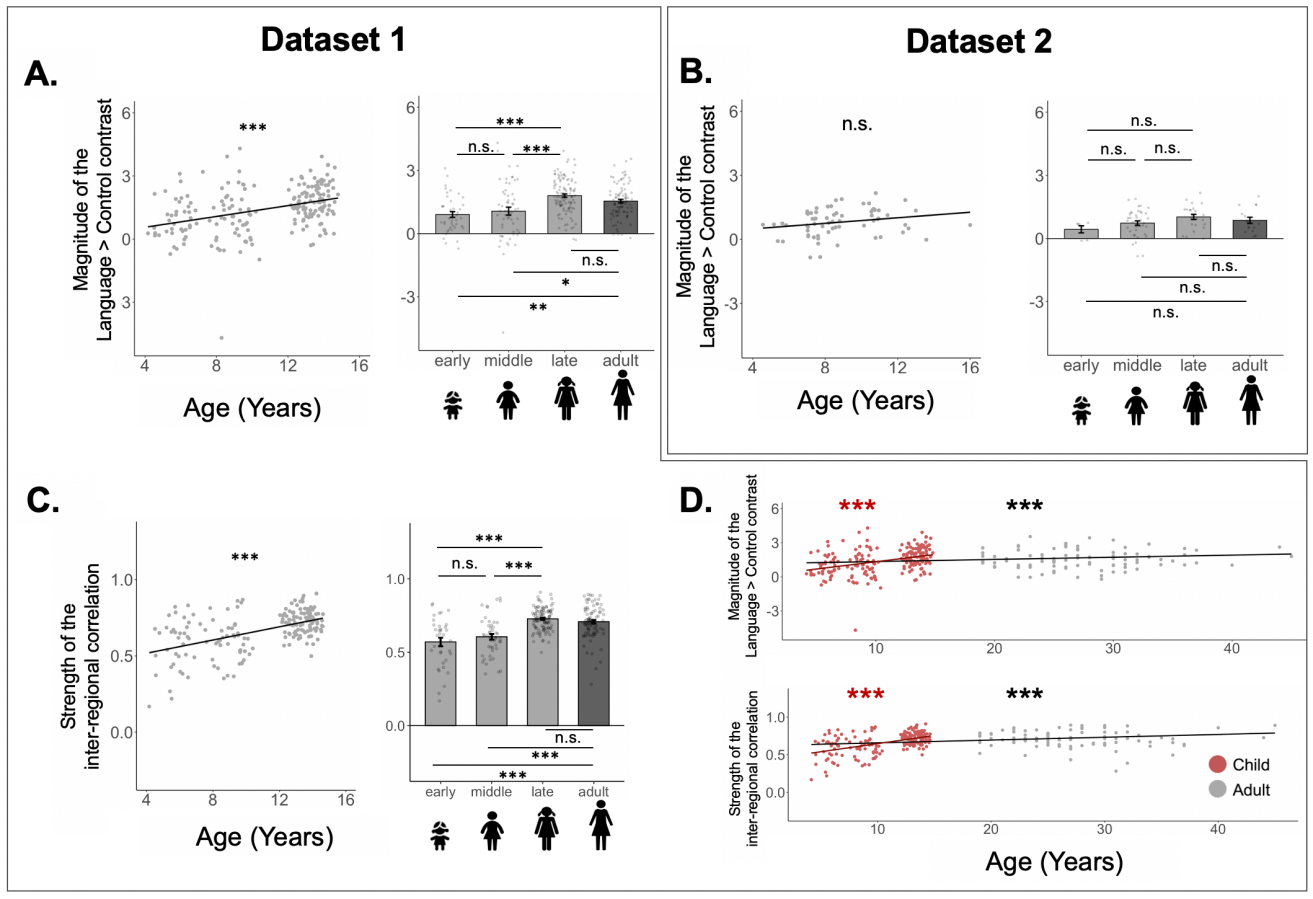
Responses to language and the strength of inter-regional functional correlations in the left hemisphere only across development. For A-C, the left column shows the results with age as a continuous variable, and the right column shows the results categorically, with a bar per age group (lighter grey bars=pediatric groups; dark grey bar=adults). **A.** Effect size (% BOLD signal change for Language>Control) in Dataset 1. **B.** Effect size for the Language>Control contrast for Dataset 2. **C.** Strength of inter-regional functional correlation (Pearson’s moment correlation) among the language regions in the left hemisphere (only available for Dataset 1). **D.** Linear age effects with magnitude of the Language>Control contrast (top) and strength of inter-regional functional correlations (bottom), with separate regression lines for child-only data (in red) and with adults included (in black). Across A-C, in the bar graphs, dots correspond to individual participants, and error bars indicate standard errors of the mean by participant; in the scatterplots, dots correspond to individual participants, and the line is the line of best linear fit. Significant effects are marked with asterisks (***=*p*<0.001; **=*p*<0.01; *=*p*<0.05; see Table 4 for details). For the bar graphs, comparisons among the pediatric groups are shown above the bars, and comparisons of each pediatric group to adults are shown below the bars.

In addition to lateralization, we examined developmental changes in overall response magnitude and strength of inter-regional correlations. These properties of the language network showed gradual development across childhood (Figure 4), as evident in both the analyses that treated age as a continuous variable and the analyses that treated age group as a categorical variable.

The analyses where age was treated as a continuous variable revealed a positive effect of age on the magnitude of the Language>Control contrast in Dataset 1 (*b* = 0.13, SE = 0.02, t = 5.66, *p* < 0.001; Figure 4A); this effect was also positive though not significant in Dataset 2 (*b* = 0.065, SE = 0.034, t = 1.91, *p* = 0.06; Figure 4). In line with these results, in Dataset 1, the Language>Control effect was larger in the late childhood and adult groups compared to the early and middle childhood groups (*ps*<0.001; Table 4A); the early and middle childhood groups did not differ significantly, and the late childhood group also did not differ from adults. In Dataset 2, the qualitative pattern was overall similar—with responses increasing from early to middle to late childhood and not showing a further increase between late childhood and adulthood—but none of the pairwise comparisons reached significance (Table 2A). This pattern of increasing response magnitude was also similar in the RH language network, across the two datasets (SI-2).

Similarly, for inter-regional correlations, the analysis where age was treated as a continuous variable revealed a positive effect of age (*b* = 0.02, SE = 0.00, t = 8.20, *p* < 0.001; Figure 4B, Table 4B). In line with this result, the correlations were stronger in the late childhood and adult groups compared to the early and middle childhood groups (*p*s<0.001; Table 4B; see SI-5 for evidence that these effects were not driven by differences related to the amount of head motion^54^); the early and middle childhood groups did not differ significantly, and the late childhood group did not differ from adults.

## Discussion

We investigated development of lateralization and overall organization of the frontal-temporal language network using two relatively large independent pediatric fMRI datasets and extensively validated language ‘localizer’ tasks^32^. Using robust individual-subject analyses, we found strong evidence for the presence of an adult-like left-hemisphere (LH) bias in the magnitude of response and volume of activation during language processing even in our youngest pediatric cohorts (4-6 year-olds), although we also found evidence that other features of the language network show a clear developmental change. In the remainder of the Discussion, we position these findings in the context of prior literature and discuss their broader implications.

### Language processing is strongly lateralized by 4 years of age and does not show a change between early childhood and adulthood.

One important claim in the literature has been that the language network starts out as a bilateral system and only becomes lateralized to the left hemisphere with age^12,13^. This hypothesis was put forward to explain a difference in the presence of language deficits following damage to the LH in mature brains vs. child brains. In particular, LH damage in adulthood typically results in aphasia^55,56^. In stark contrast, damage to the LH in children and even some adolescents can leave language largely preserved (see^16,57^ for reviews). In such cases, a typical-like language system in the homotopic right-hemisphere areas appears to support language processing^14–17,58–60^. These findings have led to arguments that RH frontal and temporal areas support language processing alongside the LH areas in developing brains, and until a certain point in the developmental trajectory can take over language function^12^.

Indeed, the very young brain shows remarkable equipotentality in response to injury or atypical experience. For example, in children born without sight, the occipital cortex responds to olfaction^61^. Thus, a distinction can be made between early equipotentiality under atypical development versus a typical, common developmental pathway for the neural networks that support language and other capacities. The question in regards to typical language development is whether or not there is long phase of bilateral response to language that slowly evolves towards a LH dominance for language.

This hypothesis makes a clear prediction that the LH and RH language areas should respond similarly to language in children, in contrast to adults, where the LH areas show stronger and more extensive responses during language processing compared to the RH areas. Several studies have indeed purported to show more bilateral responses to language in children and increasing LH-lateralization with age (e.g.,^26^; see^21^ for a review). However, we do not replicate this developmental pattern. Instead, across two large-scale datasets, we find that a) the language network is strongly lateralized already by age 4 years (complementing some earlier studies; e.g.,^28,29,31^), and b) the degree of language lateralization does not change across development. In other words, even in young children, language areas in the left hemisphere respond more strongly to language and are larger in size, compared to their RH homotopic areas. This evidence rules out the possibility that the language system is bilateral network in early childhood and becomes left-lateralized with age.

Why have some prior studies found more bilateral responses to language in childhood? Perhaps the most important contributor to these discrepancies is the use of paradigms that conflate linguistic and general task demands. In particular, many language paradigms that require overt responses from children, especially those used commonly in the clinical literature (e.g., the verb generation task^20,62^), tax both language processing and general cognitive demands associated with performing the task. Task-related cognitive demands recruit the domain-general Multiple Demand (MD) network^36,63^—a bilateral network of frontal and parietal areas whose left frontal component is adjacent to, but distinct from, the language areas^42,48,64,65^; (see^37^ for evidence that language paradigms accompanied by task demands recruit both the language and the MD network in adults). Given that most tasks are more difficult for younger children, they may recruit the MD network to a greater extent. Because the MD network is bilateral, its greater recruitment will manifest as more bilateral responses at younger ages. In other words, what looks like an increase in the degree of LH lateralization of the language network may instead reflect age-related reduction in the reliance on the (bilateral) MD network, and increased reliance on the (lateralized) language network.

We believe this issue affects an influential study by Olulade and colleagues^26^, who used a paradigm that included both linguistic demands and task demands (judging whether a sentence was true in the critical, forward-speech condition and deciding whether a tone was present at the end of the stimulus in the control, backward-speech condition), with the task in the critical condition being more difficult. When Olulade et al.’s data are preprocessed and modeled through a pipeline that helps restrict the analyses to the language areas (cf. examining activity within large anatomical masks that encompass both language and MD cortex) and when more standard lateralization measures are used—the results are similar to what we observe in the current study: no developmental change in language lateralization in children 4 years and older (SI-10).

Future work on the development of the language network should take care to separate this network from the domain-general Multiple Demand network, and other networks known to be functionally distinct from the language network, including in children^31^. One way to do this is to adopt paradigms that do not include task demands and focus on passive comprehension (e.g., contrasting passive comprehension of language with perceptually matched control conditions^32,49^). If a task is included, care should be taken to a) ensure that the task in the control condition is at least as difficult as in the critical condition, b) separate the task temporally from the language-processing component and model it separately (as in Dataset 2 in the current study), and/or c) use spatial priors for language-responsive areas based on large-scale datasets from validated paradigms (e.g.,^8^).

### Reconciling early language lateralization with early equipotentiality of the two hemispheres for language.

If language processing is already lateralized to the left hemisphere by age 4, how do we square this with evidence from early LH brain damage, which at least in some cases leaves language processing unimpaired? Existing evidence unequivocally shows that the two hemispheres *are* equipotential for language early in life (e.g.,^17,59^). Evidently, however, this equipotentiality does not manifest as similar responses in the two hemispheres to language. One possibility is that brain imaging studies, including ours, are measuring language responses too late in the developmental trajectory: maybe language processing is bilateral between age ~6 months, when linguistic abilities start to emerge^1^ and some early age: say, 1-3 years. Some evidence exists of left-hemispheric lateralization prior to age 4, but the youngest children in those cohorts (2-3 year-olds) may still be too old. Alternatively, early equipotentiality of the two hemispheres for language may be mediated by a different mechanism.

Language processing robustly recruits RH homotopic areas *across the lifespan*^8,66^ see SI-2 for evidence from the current study), although with consistently lower and less spatially extensive responses in the RH compared to the LH language areas. One reason why these RH areas can take on language function early but not later in life may be that, with age, the RH areas become specialized for storing and processing certain kinds of non-linguistic information (e.g., social information^67,68^) whereas the LH areas remain language-selective^41^.

Another possibility has to do with age-related changes in the patterns of inter-regional functional correlations. In our data, we found no hemispheric bias in the strength of inter-regional correlations during naturalistic cognition in the youngest group: similarly strong inter-regional integration in the left- and right-hemisphere language networks may at least partially underlie their equipotentiality for language early in life.

### The magnitude of response to language and the strength of inter-regional correlations increase across development and reach maturity by late childhood.

Although linguistic abilities show a high degree of sophistication by age ~4 years (e.g.,^3^), they continue to develop well into the late teens^69^. In line with this protracted developmental trajectory, two aspects of the brain organization for language processing showed a developmental change. First, the strength of the response during language comprehension relative to a control condition increased from early, to middle, to late childhood, with no difference between the late-childhood and adult groups. This pattern was highly consistent between the two datasets, despite differences in the populations tested and details of the experimental materials and paradigm (Figure 1). Second, the strength of inter-regional correlations during naturalistic cognition (resting state) showed an increase across development (including after controlling for head motion; SI-5), also reaching adult-like levels by late childhood.

Developmental changes in both the magnitude of response to language and in the strength of inter-regional correlations have been reported in past studies (e.g.,^23,25,43,70–74^). However, to the best of our knowledge, none of those studies have relied on a validated language localizer paradigm, which differentiates language areas from nearby areas of functionally distinct networks (e.g.,^47,48,65^), and on activation measures that have been established to be reliable within individuals (e.g.,^75^). Differentiating language areas from other functional areas is critical given the differences in the developmental trajectories of different cognitive abilities. For example, in contrast to linguistic abilities, which are already quite advanced by age 4-5 years, executive functions show protracted development reaching maturity in the late teens to early 20s (e.g.,^76^). Separating language and Multiple Demand areas in the current study allowed us to unambiguously attribute the neural changes we observe to the maturation of *linguistic abilities* and their underlying substrates (see also^31^).

What does the developmental increase in response magnitude to language reflect? One possibility is that it reflects relatively experience-independent biological maturation of the underlying neural circuits (e.g.,^77,78^). Another non-mutually exclusive possibility is that this increase reflects experience-related changes in linguistic ability. Our knowledge of language, which encompasses knowledge of words, constructions, and rules for combining those in new ways^79,80^, continues to expand across childhood and into adulthood. With this expansion come improvements in our ability to extract meaning from linguistic inputs. One way to distinguish between the biological vs. language-experiential accounts is to examine changes in response to language in adults as they acquire a new language. Such individuals have fully mature neural circuits but show a gradual behavioral change as they learn a language. Malik-Moraleda, Jouravlev et al.^81^ reported an investigation of polyglots—individuals with some proficiency in five or more languages—where they found that neural responses to language scale with proficiency levels. This finding provides indirect support for the idea that stronger responses in the language network reflect better linguistic ability gained through language-specific experiences, although they do not rule out a contribution of biological factors. Relating age-related neural changes to linguistic ability directly is an important avenue for future work. It is also worth noting that the magnitude of response and the strength of inter-regional correlations are only moderately correlated (r = 0.32; SI-9), which may suggest that age-related changes in these two properties are associated with distinct biological and/or cognitive changes.

### Limitations.

The current study is limited in several ways. First, we adopted a cross-sectional approach. Tracking changes in the neural infrastructure of language processing longitudinally may offer greater sensitivity in detecting age- and experience-related changes. Second, we did not attempt to match the age groups on any variables that may affect neural responses (e.g., motion, performance on language measures). This concern is somewhat ameliorated by the inclusion of two datasets and the observation of similar patterns across them; nevertheless, more carefully matched samples should be examined in future work. Third, all the pediatric data come from English, which is not representative of the world’s languages (e.g.,^82^), leaving generalization to other languages an important future direction. Finally, we have here focused on the language network—brain areas that support high-level language comprehension and production. Complementary investigations of age-related changes in lower-level speech perception areas^35,46^ and speech articulation areas^83,84^, which support the earlier-emerging abilities that scaffold language development, are another important avenue of future work.

### Open questions about the neural basis of language development.

Several important questions remain about the development of the language network. First, when and how do the language brain areas emerge? The youngest children in the current dataset are ~4 years old. This is also true of most past studies of neural language development (e.g.,^21,22,85^; cf.^31,86^). By age 4, children can understand and express complex ideas through language. What the field sorely lacks are neural data from infants and toddlers (age range: 6 months to ~3 years old). A number of studies have examined responses to speech sounds in sleeping infants during the first few days/weeks of life (e.g.,^87,88^). However, until age ~6 months^1,89^, infants do not reliably derive meaning from linguistic inputs—a core computation that the language network in adults putatively supports. Understanding how early responses to speech sounds may give rise to the language circuits a few months later remains unknown, as probing neural responses to meaningful language during the first years of life using spatially precise brain-imaging methods like fMRI poses substantial technical obstacles^90,91^.

Second, how does specialization for language processing develop and how does it relate to individual differences in language function? In adults, the brain areas that support linguistic comprehension and production are strongly specialized for language relative to diverse non-linguistic inputs and tasks, from general executive functions^5,92^, to math and logic^65,93–95^, to computer code processing^96,97^, to music^5,98^, to observing others’ actions, facial expressions, and gestures^99–101^, to social cognition^68^. How does this specialization emerge? Do brain areas that selectively support language in adults perform different or additional functions earlier in life? The fact that the language system is closely juxtaposed with other large-scale brain networks^42,48,77^ may suggest that at earlier points in development (and perhaps evolution) the language system is not segregated from some of these nearby areas. Hiersche et al.^31^ suggest that by age ~3 years, the language system is robustly segregated from the domain-general Multiple Demand network, which has been linked to fluid reasoning and general intelligence. However, testing other non-linguistic functions relative to language processing, and at earlier ages, will be critical.

Third, we, as a field, still lack an understanding of what is special about the left hemisphere: i.e., why in most typical individuals does language processing end up being localized to the left? Aso, why does language processing appear to be more bilateral in many individuals with developmental brain disorders^102,103^ (see^104^ for a meta-analysis) and in some other populations with atypical developmental experiences, like congenitally blind individuals^105^? Hypotheses about the left-hemisphere bias for language are plentiful (e.g.,^106^), but getting a clear answer has proven challenging. Understanding whether language processing is already lateralized in infants and/or toddlers may place some constraints on this hypothesis space.

In conclusion, we have shown that the human language system is strongly lateralized to the left hemisphere even in young children, which challenges the hypothesis that the language system is bilateral in childhood^12,13^. Although the level of response to language and the inter-connectedness of the language network continues to increase into late childhood, adult-like lateralization to the left hemisphere is already present by age 4-5 years.

## Methods

### 1. Participants

For all child participants, a parent or guardian provided written informed consent, and the child provided assent; adult participants provided written informed consent, in accordance with the Committee on the Use of Humans as Experimental Subjects at the Massachusetts Institute of Technology.

#### Inclusion criteria:

Across datasets, child and adult participants were included in the analysis based on the following criteria: 1) being a native speaker of the language used in the experiment (English for all groups except for the adult group in Dataset 1; diverse languages for the adult group in Dataset 1, as described in^40^), 2) having no diagnosis of neurological disorder, and 3) having normal or corrected-to-normal vision. For Dataset 1, it was additionally ensured that child participants were born full-term (>37 weeks), had no history of brain injury and hospitalizations, and were not using psychotropic medications.

#### Dataset 1:

After excluding 28 child participants from group-level analyses due to excessive motion (Methods-Section5; for summary of motion outliers by group, see https://osf.io/vxe8d), the final Dataset 1 included 206 children (ages 4-14; average = 10.69, st. dev. = 3.18; 106 females; 178 right-handed) and 91 adults (ages 19-45, average = 27.40, st. dev. = 5.5; 48 females; 84 right-handed). The pediatric data came from three projects that each focused on a different age range and did not concern the development of the language network (for details, see https://osf.io/3mvpx/?view_only=803ba79e635d4e0da1698e0a6367d7e6): early childhood (n=41; ages 4-6, average = 5.78, st. dev. = 0.69), middle childhood (n=57; ages 7-10, average = 9.00, st. dev. = 0.74), and late childhood (n=108; ages 12-14, average = 13.45, st. dev. = 0.69).

#### Dataset 2:

After excluding 31 child participants and 0 adult participants from group-level analyses due to excessive motion (Methods-Section5; for summary of motion outliers by group, see https://osf.io/vxe8d), the final Dataset 2 included 67 children (ages 4-16; average = 11.5, st. dev. = 1.4; 29 females) and 16 adults (ages 18-60, average = 43.8, st. dev. = 12.1; 7 females). To enable parallel analyses across the two datasets, the pediatric data in this dataset were divided into three age groups, to approximately match the division in Dataset 1: early childhood (n=6; ages 4-6, average = 5.4, st. dev. = 0.46), middle childhood (n=38; ages 7-9, average = 7.8, st. dev. = 0.65), and late childhood (n=23; ages 10-16 average = 11.5, st. dev. = 1.38).

A subset of the children and adults in Dataset 2 wore a total light-exclusion blindfold during the fMRI scan to reduce visual cortex responses to visual input during the scan, for a different study^107^ (children: n=14 [early = 2; middle = 7; late = 5], ages 4-16, average =8.85 , st. dev. = 2.23; adults: n=16, ages 18-59, average = 43.8, st. dev. =12.08). Blindfolded and non-blindfolded children are treated as a single group for the current study, which focuses on responses in higher-cognitive networks.

Subsets of Datasets 1 and 2 were included in several published studies^40,105,108–110^), whose goals differed from the current study and did not concern the development of the language network.

### 2. Language localizer task

As shown in Figure 1, four variants of the language localizer were used, with the control condition varying between speech played in reverse, acoustically degraded speech, and speech in an unfamiliar foreign language (see SI-1 for details). Each participant completed 6-12 blocks per condition across 1-4 scanning runs (for the early childhood group in Dataset 1, where a single run was used, the run was manually split into two equal-sized runs to enable across-runs cross-validation, as needed for the analyses; see Critical individual-level neural measures). Participants were excluded from the language localizer task analysis if more than 40% of the acquired volumes (across both runs) were identified as outlier volumes during preprocessing (as detailed below).

In addition to the language localizer task, most participants in Dataset 1 performed a resting state scan (described below) and most participants performed additional tasks that were unrelated to the current study.

### 3. Resting state scan

The majority of participants in Dataset 1 performed a resting state scan. The participants in the early childhood group were asked to keep their eyes open and to think about anything that came to mind. A fixation cross was shown on the screen, but the children were not instructed to keep their eyes on it. The scan lasted 5 min 15 s. The participants in the middle and late childhood groups were asked to keep their eyes on the fixation cross and to let their mind wander. The scan lasted 6 min and 22 s for the Middle Childhood group and 5 min and 46 s for the Late Childhood group. For the Adult group, following^64^, participants were asked to close their eyes but to stay awake and to let their mind wander; the projector was turned off, and the lights were dimmed. The scan lasted 5 min.

Participants were excluded from the resting state analysis if more than 40% of the acquired resting state volumes were identified as outlier volumes during preprocessing (as detailed below). Of the 206 and 91 adults that were included in the language localizer analyses, 189 children (ages 4 – 14, average = 10.93, st. dev. = 3.15, 97 females) and 90 adults (ages 19-45, average = 27.38, st. dev. = 5.54, 47 females) were included in the resting state analyses (n=6 early and n=14 middle participants were excluded because of excessive motion). The pediatric participants included: early childhood (n=35; ages 4-6, average = 5.78, st. dev. = 0.69), middle childhood (n=47; ages 7-10, average = 9.04, st. dev. = 0.78), and late childhood (n=107; ages 12-14, average = 13.44, st. dev. = 0.68).

### 4. fMRI data acquisition

All data were collected on a 3-Tesla Siemens Prisma scanner at the Athinoula A. Martinos Imaging Center at the McGovern Institute for Brain Research at MIT.

#### Dataset 1:

##### Early Childhood:

Whole-head, high-resolution T1-weighted multi-echo MPRAGE structural images were acquired in 176 sagittal slices (TR = 2,530 ms, TE = 1.64 ms/3.5 ms/5.36 ms/7.22 ms, TI = 1,400 ms, flip angle = 7°, resolution = 1.00 mm isotropic). Whole-brain functional blood oxygenation level dependent (BOLD) data were acquired using an echoplanar imaging (EPI) T2*-weighted sequence in 41 transverse slices (2 mm thick) in an interleaved order (TR = 2,500 ms, TE = 30 ms, flip angle = 90°, bandwidth = 2,298 Hz/Px, echo spacing = 0.5 ms, FoV = 192 mm, phase encoding A > P direction, in-plane resolution = 3 mm × 3 mm). Resting state scans were acquired using an EPI T2*-weighted sequence in 41 sagittal slices (3 mm thick) in an interleaved order with a 10% distance factor (TR = 520 ms, TE 1 = 3.41 ms, TE 2 = 5.97 ms, flip angle = 55°, bandwidth = 1,502 Hz/Px, FoV = 192 mm, phase encoding A > P direction, in-plane resolution = 3 mm x 3 mm). Prospective acquisition correction (PACE) was used to adjust the position of the gradients based on the participant’s motion one TR back.

##### Middle Childhood:

Whole-head, high-resolution T1-weighted multi-echo MPRAGE structural images were acquired in 176 sagittal slices (TR = 2,530 ms, TE = 1.69 ms/3.55 ms/5.41 ms/7.27 ms, TI = 1,400 ms, flip angle = 7°, resolution = 1.00 mm isotropic). Whole-brain functional BOLD data were acquired using an EPI T2* weighted sequence in 32 near-axial slices (4 mm thick) in an interleaved order with a 10% distance factor (TR = 2,000 ms, TE = 30 ms, flip angle = 90°, bandwidth = 1,860 Hz/Px, echo spacing = 0.63 mm, FoV = 200 mm, phase encoding A > P direction, in-plane resolution = 2.1 mm x 2.1 mm). Resting state scans were acquired using an EPI T2*-weighted sequence in 40 sagittal slices (3 mm thick) in an interleaved order with a 10% distance factor (TR = 2,500ms, TE = 30 ms, flip angle = 90°, bandwidth = 2,380 Hz/Px, echo spacing = 0.49 mm, FoV = 210 mm, phase encoding A > P direction, in-plane resolution = 3 mm x 3 mm). PACE was used to adjust the position of the gradients based on the participant’s motion one TR back^111^.

##### Late Childhood:

Whole-head, high-resolution T1-weighted multi-echo MPRAGE structural images were collected in 320 sagittal slices (TR = 4,000 ms, TE = 1.06 ms, flip angle = 2°, resolution = 2.00 mm isotropic). Whole-brain functional BOLD data were acquired using an EPI T2*-weighted sequence in 72 near-axial slices (4 mm thick) in an interleaved order with a 10% distance factor and using GRAPPA with an acceleration factor of 2 (TR = 1,000 ms, TE = 37.2 ms, flip angle = 63°, bandwidth = 2,290 Hz/Px, echo spacing = 0.58 mm, FoV = 208 mm, phase encoding A > P direction, matrix size = 96 × 96, in-plane resolution = 2 mm x 2 mm). Resting state scans were acquired using an EPI T2*-weighted sequence in 72 axial slices (2 mm thick) in an interleaved order with a 0% distance factor (TR = 800 ms, TE = 37 ms, flip angle = 52°, bandwidth = 2,290 Hz/Px, echo spacing = 0.58 mm, FoV = 208 mm, phase encoding A > P direction, in-plane resolution = 2 mm x 2 mm).

##### Adults:

Whole-head, high-resolution T1-weighted multi-echo MPRAGE structural images were collected in 179 sagittal slices (TR = 2,530 ms, TE = 3.48 ms, resolution = 1 mm isotropic). Whole-brain functional BOLD data were acquired using an EPI T2*-weighted sequence in 31 near-axial slices (4 mm thick) in an interleaved order with a 10% distance factor and using GRAPPA with an acceleration factor of 2 (TR = 2,000 ms, TE = 30 ms, flip angle = 90°, bandwidth = 1,578 Hz/px, echo spacing = 0.72 ms, FoV = 200 mm, phase encoding A > P direction, matrix size = 96 x 96, in-plane resolution = 2.1 mm x 2.1 mm).

#### Dataset 2:

Whole-head, high-resolution T1-weighted structural images were collected using one of two custom 32-channel head coils made for children^112^, a 12-channel coil, or the standard Siemens 32-channel head coil in 176 interleaved sagittal slices (TR = 2,530 ms, TE= 1.64/3.5 ms/5.36 ms/7.22 ms, resolution = 1 mm isotropic). Whole-brain functional BOLD data were collected in 32 near-axial slices (3 mm thick) in an interleaved order with a 20% distance factor (TR = 2,000 ms, TE = 30 ms, flip angle = 90°, bandwidth = 2298 Hz/Px, custom pediatric coils: FoV = 192 mm; standard 32-channel head coil: FOV = 256 mm; 12-channel head coil: FOV = unavailable, phase encoding A>P direction, matrix size = 64x64, in-plane resolution = 3 mm × 3 mm). PACE was used to adjust the position of the gradients based on the participant’s motion one TR back^111^.

### 5. fMRI data preprocessing and first-level modeling

fMRI data were preprocessed and analyzed using SPM12 (release 7487), CONN EvLab module (release 19b) and custom MATLAB scripts. Each participant’s functional and structural data were converted from DICOM to NIFTI format. All functional scans were co-registered and resampled using B-spline interpolation to the first scan of the first session. Potential outlier scans were identified from the resulting subject-motion estimates as well as from BOLD signal indicators using default thresholds in the CONN pre-processing pipeline (5 st. dev. or more above the mean in global BOLD signal change or framewise displacement values above 0.9 mm). Functional and structural data were independently normalized into a common space (the Montreal Neurological Institute (MNI) template, IXI549Space) using the SPM12 unified segmentation and normalization procedure with a reference functional image computed as the mean functional image after realignment across all time points, omitting outlier scans. The output data were resampled to a common bounding box between MNI-space coordinates (−90, −126, and −72) and (90, 90, and 108), using 2 mm isotropic voxels and fourth-order spline interpolation for the functional data and 1 mm isotropic voxels and tri-linear interpolation for the structural data. Lastly, the functional data were smoothed spatially using spatial convolution with a 4 mm full-width half-maximum (FWHM) Gaussian kernel.

For the critical and control conditions of the language localizer task, effects were estimated using a general linear model (GLM) in which each experimental condition was modeled with a boxcar function convolved with the canonical hemodynamic response function (HRF) (fixation was modeled implicitly). Temporal autocorrelations in the BOLD signal timeseries were accounted for by a combination of high-pass filtering with a 128 s cutoff and whitening using an AR (0.2) model (first-order autoregressive model linearized around the coefficient a = 0.2) to approximate the observed covariance of the functional data in the context of restricted maximum likelihood (ReML) estimation. In addition to main condition effects, other model parameters in the GLM design included first-order temporal derivatives for each condition (for modeling spatial variability in the HRF delays) as well as nuisance regressors to control for the effect on the BOLD signal of slow linear drifts, subject-motion parameters, and outlier scans.

The resting state data were pre-processed using the CONN toolbox with default parameters unless stated otherwise. First, to remove noise resulting from signal fluctuations originating from non-neuronal sources (for example, cardiac or respiratory activity), the first five BOLD signal time points extracted from the white matter and cerebrospinal fluid (CSF) were regressed out of each voxel’s time course. White matter and CSF voxels were identified based on segmentation of the anatomical image^113^. Second, the residual signal was band-pass filtered at 0.008–0.09 Hz to preserve only low-frequency signal fluctuations^114^.

### 6. Functional ROI (fROI) definition

For each participant, functional regions of interest (fROIs) were defined using the Group-constrained Subject-Specific (GcSS) approach^32^. For the language network in the left hemisphere (LH), we used five parcels derived from a group-level representation of the language localizer data in 220 adult participants (independent of the adult sample in the current study) and used in much past work (e.g.,^8,51,68,96,100,103,115,116^, *inter alia*). These parcels include three regions in the left frontal cortex (two in the inferior frontal gyrus (LIFG and LIFGorb) and one in the middle frontal gyrus (LMFG)) and two regions in the left temporal cortex (LAntTemp and LPostTemp). Individual fROIs were defined by selecting—within each parcel—the 10% of most localizer-responsive voxels based on the *t*-values for the Language>Control contrast (see^8^ for evidence that fROIs defined in this way are similar to fROIs based on a fixed statistical significance threshold). We additionally defined a set of language-responsive areas in the right hemisphere (RH). Following past work (e.g.,^40^), we projected the LH parcels onto the right hemisphere and selected the 10% of most localizer-responsive voxels, as in the LH. (We chose to use parcels derived from adults in order to be able to directly compare critical neural measures between children and adults, but see SI-6 for evidence that parcels derived from the pediatric data directly are similar.)

### 7. Critical individual-level neural measures of language processing

Statistical analyses were performed on a set of individual-level neural measures of language processing, including i) the magnitude of neural response, ii) the volume of activation, and iii) the strength of inter-regional functional correlations during naturalistic cognition (resting state).

#### Response magnitude:

We extracted the responses (in percent BOLD signal change) from each individually defined language fROI (averaging the responses across the voxels in each fROI) to each condition (Language (Intact/Forward) and Control (Degraded/Backward/Foreign language)) relative to the fixation baseline. To ensure independence between the data used to define the fROIs and to estimate their response magnitudes, we used an across-runs cross-validation procedure (e.g.,^33^). Response magnitude was averaged across run splits, resulting in one value per particpant for statistical analyses.

#### Volume of activation:

Following past work (e.g.,^40,75,103^), we extracted the total number of significant voxels for each fROI above the uncorrected p<0.001 for DS1 and p<0.01 for DS2 threshold for Language>Control contrast. At these thresholds, most participants showed suprathreshold voxels.

#### Inter-region functional correlation:

For the participants with resting state data, we extracted the BOLD signal timeseries from each individually defined language fROI (averaging the responses across the voxels in each fROI) during the resting state scan. We then computed Pearson’s moment correlation coefficients between the timeseries for each pair of fROIs (45 pairwise correlations among the 10 language fROIs, 5 fROIs in each hemisphere). These correlations were Fisher-transformed to improve normality and decrease biases in averaging^117^.

#### Lateralization:

To determine the degree of LH-lateralization, we used the following formula to calculate the lateralization index for each of the five bilateral language regions: (LH − RH) / (LH + RH) for the response magnitude, volume of activation, and inter-regional functional correlation measures. For response magnitude, we use the t-values^118^ for the Language>Control contrast averaged across the voxels in each fROI and then across the five fROIs for each hemisphere in each participant. We used t-values to minimize the number of participants with negative contrast values; even so, a few participants showed negative average t-values in one or both hemispheres; to be able to compute the LI values, we used a standard approach of baseline correction, where the largest negative value was added to each value separately for DS1 and DS2^119^. For volume, the total number of significant voxels per each parcel was used. For the inter-regional functional correlation, the LH and RH values represented the mean correlation among all fROI pairs in each hemisphere.

### 8. Statistical Analyses

We asked three research questions about the development of the language network, as described next. The analyses were identical across the two datasets, except for analyses of inter-region functional correlations, which were only performed for Dataset 1.

#### 1) Do children (of different ages) show adult-like topography of the left hemisphere language network?

Do children show a reliable response to language relative to the control condition in the LH language network overall, and in the temporal and frontal components separately? For each age group in each dataset, we fit a linear mixed-effects regression model predicting the BOLD response (estimated in each region as described in Critical individual-level neural measures) from condition (Language vs. Control) with random intercepts for participants and fROIs (all of the analyses, including model specification, are available at: https://osf.io/j582b). For completeness, we additionally fit the same model for the RH homotope of the language network (SI-2).

#### 2) Do children (of different ages) show adult-like left hemispheric bias?

First, we tested the effects of hemisphere on the three critical neural measures: i) Magnitude of the Language>Control contrast extracted as described above; ii) Volume of activation for the Language>Control contrast extracted by calculating for each participant in each dataset, the number of language-responsive voxels summed across all parcels, the temporal parcels, and the frontal parcels in each hemisphere. Based on the examination of individual whole-brain activation maps, we chose the p<0.001 uncorrected whole-brain threshold for Dataset 1 and the p<0.01 uncorrected whole-brain threshold for Dataset 2 (the activations were generally weaker for Dataset 2); iii) Strength of inter-regional correlations in the language network was obtained by extracting average pairwise within-hemisphere correlations as described above.

For each of these three critical measures, we fit a linear mixed-effects regression model to test for the effects of age. In one set of analyses, we treated age as a continuous variable and focused on the pediatric population; in a complementary set of analyses, we treated age group (early, middle, late childhood, and adult) as a categorical variable. For the latter analyses, we compared each pediatric group to the adult group using mixed-effects regression models that include age group as a fixed effect. additionally compared the three pediatric groups to each other. We additionally fit the same model separately for the temporal and frontal components.

Next, we asked whether/how the different properties of the language network change over the course of development, controlling for absolute differences in the magnitude of effects and potential task confounds using the lateralization index following prior work (e.g.,^40,75,103,120^). In particular, we used the following formula on the LH and RH network Language>Control effects, volumes of activation, and inter-regional correlation coefficients obtained as described above: (LH − RH) / (LH + RH); the resulting values vary from 1 (exclusively LH activations) to −1 (exclusively RH activations).

We examined the effect of age on the LIs based on magnitude, volume, and inter-regional correlation, both continuously and categorically. For magnitude values, there were two subjects with negative values (one in DS1 and one in DS2). To allow for LI calculation, we transformed the mean values for each hemisphere by adding the absolute value of the lowest negative number across all fROIs to all values separately for DS1 and DS2. We then fit a linear regression model predicting each LI measure (based on magnitude, volume, and inter-regional correlation) from age as a continuous variable. We then re-ran the linear regressions with age as a categorical variable.

#### 3) Are there age-related differences in the magnitude of response and inter-regional correlations?

To better understand developmental changes in other properties of the language regions, we evaluated the effects of age on (1) the magnitude of activation in the Language>Control contrast and (2) the strength of inter-regional correlations within the language network during resting state. We fit linear mixed-effects models to predict both effects from age (with separate models using age as a continuous and categorical variable), with participants and fROIs as random effects. For the categorical analysis, we then performed pairwise comparisons of the adjusted means for age using Tukey’s method for multiple comparison adjustments and to examine the direction of effects.

All analyses were performed in R v3.5.0 (R Core Team, 2013), using identical statistical thresholds (p < 0.05), and random effect structures (using the package lme4^121^. Significance of fixed effects in the models was tested in an ANOVA and fitted with restricted maximum likelihood (REML) using the package lmerTest^122^. Degrees of freedom were estimated using the Satterthwaite approximation^122,123^.

In addition to the analyses reported in the main text, we performed a version of the analyses where the total number of outlier volumes (see fMRI data preprocessing and first-level modeling) was used as a covariate. The results of the analyses are reported in SI-5 The patterns of results were not affected by the inclusion of this covariate.

## Supplementary Material

Supplement 1

## Figures and Tables

**Table 1.** Magnitude of responses to language in the left hemisphere (LH) language network as a whole, and in the temporal and frontal components separately, across development. For each age group [DS1 n=41; DS2 n=6], middle childhood [DS1 n=57; DS2 n=38], late childhood [DS1 n=108; DS2 n=23], and adulthood [DS1 n=91; DS2 n=16]) in each dataset (DS1, DS2), we fit a linear mixed-effects regression model predicting the magnitude of the BOLD response (estimated in each region as described in Methods-Section7) from condition (Language vs. Control) with random intercepts for participants and fROIs (Methods-Section8). The *p*-values for the temporal and frontal areas are uncorrected, but all survive a Bonferroni correction for two comparisons.

Magnitude of responses to the Language>Control contrast													
		Language network				Temporal language areas				Frontal language areas			
		*b*	*SE*	*t*	*p*	*b*	*SE*	*t*	*p*	*b*	*SE*	*t*	*p*
DS 1	Early	0.91	0.08	11.2	**< 0.001**	1.58	0.18	8.61	**< 0.001**	0.77	0.11	7.06	**< 0.001**
	Middle	1.05	0.13	8.42	**< 0.001**	1.55	0.19	7.96	**< 0.001**	0.72	0.13	5.72	**< 0.001**
	Late	1.81	0.06	32.85	**< 0.001**	2.53	0.079	32.15	**< 0.001**	1.32	0.07	20.01	**< 0.001**
	Adult	1.54	0.06	24.88	**< 0.001**	1.78	0.11	25.02	**< 0.001**	1.34	0.07	20.01	**< 0.001**
DS 2	Early	0.44	0.1	4.58	**< 0.001**	0.56	0.16	3.41	**0.01**	0.35	0.1	3.54	**< 0.01**
	Middle	0.73	0.06	11.75	**< 0.001**	0.84	0.09	9.2	**< 0.001**	0.66	0.08	8.02	**< 0.001**
	Late	1.03	0.07	14.34	**< 0.001**	1.1	0.09	11.78	**< 0.001**	0.97	0.1	10.05	**< 0.001**
	Adult	0.86	0.1	9.04	**< 0.001**	0.81	0.11	7.45	**< 0.001**	0.9	0.14	6.53	**< 0.001**

**Table 2.** A LH bias in response to language and the strength of inter-regional functional correlations in the language network across development. **A. Effect of hemisphere on the magnitude of the Language>Control contrast.** For each age group in each dataset, we fit a linear mixed-effects regression model predicting the average effect size (averaged across regions; including random intercepts for fROIs led to the model not converging; effects sizes were estimated as described in Methods-Section7) from hemisphere with random intercepts for participants. **B. Effect of hemisphere on the volume of activation for the Language>Control contrast.** For each age group in each dataset, we fit a linear mixed-effects regression model predicting the total number of voxels (across regions; number of voxels was calculated as described in Methods-Section7) from hemisphere with random intercepts for participants. **C. Effect of hemisphere on the strength of inter-regional correlations in the language network.** For each age group in Dataset 1, we fit a linear mixed-effects regression model predicting average pairwise inter-region correlation (correlations were estimated as described in Methods-Section 7) from hemisphere with random intercepts for participants.

A. Effect of hemisphere on the magnitude of the Language>Control contrast					
		*b*	*SE*	*t*	*p*
DS 1	Early	0.75	0.19	3.90	**< 0.001**
	Middle	0.30	0.22	1.37	0.17
	Late	0.66	0.11	6.14	**< 0.001**
	Adult	0.48	0.12	4.07	**< 0.001**
DS 2	Early	0.24	0.28	0.88	0.40
	Middle	0.29	0.14	2.06	**0.04**
	Late	0.59	0.17	3.50	**0.001**
	Adult	0.52	0.16	3.14	**0.004**
B. Effect of hemisphere on the volume of activation for the Language>Control contrast					
		*b*	*SE*	*t*	*p*
DS 1	Early	272.2	88.83	3.06	**0.004**
	Middle	425.21	90.4	4.7	**< 0.001**
	Late	1087.91	87.3	12.46	**< 0.001**
	Adult	614.09	79.32	7.74	**< 0.001**
DS 2	Early	285	202.84	1.41	0.22
	Middle	250.58	90.29	2.78	**0.01**
	Late	656.65	185.64	3.54	**0.002**
	Adult	680.9	119.9	5.68	**<0.001**
C. Effect of hemisphere on the strength of inter-regional correlations in the language network					
		*b*	*SE*	*t*	*p*
DS1	Early	0	0.03	0.06	0.95
	Middle	0.05	0.02	2.79	**0.008**
	Late	0.04	0.01	5.31	**< 0.001**
	Adult	0.04	0.01	3.44	**< 0.001**

**Table 3.** Stability of LH bias in response to language and the strength of inter-regional functional correlations across age groups. For each participant in each age group, we computed the Lateralization Index (LI) for each measure using the formula (LH effect − RH effect) / (LH effect + RH effect) averaged across fROIs. LI measures control for overall effect sizes, allowing to differentiate lateralization differences from possible confounding factors. We then performed two kinds of analyses: continuous and categorical. **A. Effect of age on the magnitude of the Language>Control contrast.** Mean effects for each hemisphere were extracted from the top 10% (most active) voxels for each fROI and averaged across fROIs before calculating LI. For all participants across the three childhood groups, we fit a linear regression model predicting the LI based on the magnitude of the response from age as a continuous variable. We then reran the linear regression model using age group as a categorical predictor, followed by inter-group pairwise comparisons adjusted using Tukey’s method. **(B) Effect of age on the volume of activation for the Language>Control contrast.** For all participants across the three childhood groups, we fit a linear regression model predicting the LI based on the volume of activation from age as a continuous variable, with random intercepts for participants and fROIs. We then reran the linear regression model using age group as a categorical predictor, with random intercepts for participants and fROIs, followed by inter-group pairwise comparisons adjusted using Tukey’s method. **(C) Effect of age on the strength of inter-regional correlations.** For all participants across the three childhood groups, we fit a linear regression model predicting the LI based on the inter-regional correlation measure from age as a continuous variable. We then reran the linear regression model using age group as a categorical predictor, followed by inter-group pairwise comparisons adjusted using Tukey’s method.

A. Age differences in lateralization for the magnitude of the Language>Control contrast					
		*b*	*SE*	*t*	*p*
DS 1	Age = Continuous (Ages 4-16)	0.00	0.00	1.29	0.20
	Early vs. Adult	0.06	0.04	1.65	0.35
	Middle vs. Adult	−0.06	0.03	−1.82	0.26
	Late vs. Adult	0.06	0.03	2.07	0.17
	Early vs. Middle	0.13	0.04	3.02	**0.02**
	Middle vs. Late	−0.12	0.03	−3.67	**0.002**
	Early vs. Late	0.00	0.04	0.09	1.00
DS 2	Age = Continuous (Ages 4-16)	−0.00	0.00	1.25	0.22
	Early vs. Adult	0.12	0.11	1.07	0.71
	Middle vs. Adult	−0.13	0.07	−1.9	0.25
	Late vs. Adult	−0.03	0.08	−0.37	0.98
	Early vs. Middle	0.25	0.10	2.44	0.08
	Middle vs. Late	−0.10	0.06	−1.67	0.35
	Early vs. Late	0.15	0.11	1.38	0.52
B. Age differences in lateralization for the volume of activation for the Language>Control contrast					
		*b*	*SE*	*t*	*p*
DS 1	Age = Continuous (Ages 4-16)	−0.01	0.01	−0.71	0.48
	Early vs. Adult	−0.16	0.08	−1.97	0.2
	Middle vs. Adult	0.11	0.06	1.52	0.43
	Late vs. Adult	0.01	0.07	0.18	1.00
	Early vs. Middle	0.27	0.09	2.82	**0.01**
	Middle vs. Late	0.12	0.07	1.62	0.24
	Early vs. Late	0.15	0.08	1.72	0.19
DS 2	Age = Continuous (Ages 4-16)	−0.01	0.03	−0.55	0.59
	Early vs. Adult	−0.12	0.22	−0.52	0.95
	Middle vs. Adult	−0.12	0.13	−0.89	0.89
	Late vs. Adult	−0.11	0.14	−0.66	0.91
	Early vs. Middle	0.01	0.20	0.04	1.00
	Middle vs. Late	−0.01	0.12	−0.11	1.00
	Early vs. Late	−0.01	0.21	−0.03	1.00
C. Age differences in lateralization for the strength of inter-regional correlations					
		*b*	*SE*	*t*	*p*
DS 1	Age = Continuous (Ages 4-16)	0	0	0.84	0.40
	Early vs. Adult	−0.02	0.02	−1.15	0.66
	Middle vs. Adult	0.02	0.02	1.09	0.69
	Late vs. Adult	0.00	0.01	0.17	1.00
	Early vs. Middle	−0.04	0.02	−1.90	0.23
	Middle vs. Late	0.02	0.02	0.98	0.76
	Early vs. Late	−0.02	0.02	−1.30	0.56

**Table 4.** Age-related changes in the magnitude of the Language>Control contrast in the LH language network (A) and the strength of inter-regional correlations among LH language fROIs (B). We conducted linear mixed-effects analyses predicting response magnitude and IRC strength of the language network from Age (categorical and continuous) with random intercepts for participants and fROIs. We then performed pairwise comparisons of the adjusted means for Age using Tukey’s method for multiple comparison adjustments. **A.** We estimated Language>Control BOLD responses in each region (as described in Methods-Section7) for each participant. **B.** For each age group in Dataset 1 we calculated pairwise inter-region resting-state correlations among all left-hemisphere fROIs (Language Network).

A. Age differences in the magnitude of the Language>Control contrast					
		*b*	*SE*	*t*	*p*
Dataset 1	Age = Continuous (Ages 4-16)	0.13	0.02	5.66	**< 0.001**
	Early vs. Adult	−0.64	0.18	−3.46	**0.004**
	Middle vs. Adult	−0.48	0.16	−2.81	**0.02**
	Late vs. Adult	0.26	0.14	1.88	0.24
	Early vs. Late	−0.9	0.19	−4.72	**< 0.001**
	Middle vs. Late	−0.75	0.16	−4.57	**< 0.001**
	Early vs. Middle	−0.16	0.21	−0.74	0.74
Dataset 2	Age = Continuous (Ages 4-16)	0.065	0.034	1.91	0.06
	Early vs. Adult	0.43	0.29	1.47	0.46
	Middle vs. Adult	0.13	0.18	0.72	0.89
	Late vs. Adult	0.16	0.2	0.83	0.84
	Early vs. Middle	−0.3	0.27	−1.1	0.52
	Middle vs. Late	−0.29	0.16	−1.82	0.17
	Early vs. Late	−0.59	0.28	−2.11	0.097
B. Age differences in the strength of inter-regional correlations					
		*b*	*SE*	*t*	*p*
Dataset 1	Age = Continuous (Ages 4-16)	0.02	0.00	8.20	**< 0.001**
	Early vs. Adult	−0.14	0.02	−6.14	**< 0.001**
	Middle vs. Adult	−0.1	0.1	−5.08	**< 0.001**
	Late vs. Adult	0.02	0.02	1.16	0.65
	Early vs. Middle	−0.03	0.03	−1.34	0.36
	Middle vs. Late	−0.12	0.02	−6.14	**< 0.001**
	Early vs. Late	−0.16	0.02	−7.1	**< 0.001**
