# Supplementary material for: Precision fMRI reveals that the language network exhibits adult-like left-hemispheric lateralization by 4 years of age
Source: bioRxiv. 2024 Jun 12:2024.05.15.594172. Originally published 2024 May 15. Preprint. [Version 2] doi: 10.1101/2024.05.15.594172 (PMC11118489; doi:10.1101/2024.05.15.594172)
Supplement: Supplementary file 1 [file media-1.pdf]

Supplementary Information for Ozernov-Palchik, O'Brien et al., "Precision fMRI reveals that the language network exhibits adult-like left-hemispheric lateralization by 4 years of age"

Table of Contents

SI-1: A detailed description of the language localizer paradigm variants.

SI-2: Magnitude of responses to language in the RH language network across development. *(parallel to Results section 1)*

SI-3: Effect of hemisphere on the volume of activation for the Language > Control contrast in the temporal and frontal components of the language network across development. (*parallel to Results section 2, first half)*

SI-4: Strength of inter-regional correlations among the LH (A) and RH (B) language fROIs across development. *(complementing IRC results in Results section 2)*

SI-5: Age-related changes in the magnitude of the Language > Control contrast in the LH language network and the strength of inter-regional correlations among LH language fROIs, controlling for motion. *(parallel to Results section 3)*

SI-6: Group-constrained Subject-Specific (GSS) analysis of the pediatric participants.

SI-7: Within- and between-participant correlations of the fine-grained activation patterns for the Language > Control contrast in Dataset 1.

SI-8: Correlations between the Language > Control magnitude in the LH language network and the strength of inter-regional correlations among the LH language fROIs in Dataset 1.

SI-9: A systematic comparison between the data from the current study and the data and analyses from Olulade et al.'s (2020) study with respect to the lateralization of the language network.

SI-1: A detailed description of the language localizer paradigm variants.

Localizer Variant 1 (used for the early childhood group in Dataset 1): The critical condition consisted of short (15 s long) stories from the '*Narrative Language Measures'* component of the CUBED language assessment (Peterson & Spencer, 2012). The stories were recorded by a female native speaker of English and described events that children in this age range are likely to be familiar with (e.g., playing games, getting hurt). The control condition consisted of the same clips played in reverse (see e.g., Bedny et al., 2011 for a similar approach). (A third condition, of no relevance for the current study, included different critical stimuli played to different ears.) The materials used in this variant, along with Variants 2 and 3 are available on OSF: https://osf.io/3mvpx/. Participants were instructed to listen to the clips, and a picture of a female stick figure appeared on a gray screen throughout auditory stimulation in both conditions to remind children to listen. Each participant performed one run, which consisted of 6 experimental events for each of the three conditions and 18 fixation periods (5 s each), for a total run duration of 360 s (**Table SI-1**). Condition order was pseudo-randomized for each participant, so that no events from the same condition appeared in a row. (The runs were manually divided into two runs for analyses, as described in Methods-Section2.)

Localizer Variant 2 (used for the middle childhood and adult groups in Dataset 1): The critical condition consisted of short (18 s long) passages from *Alice's Adventures in Wonderland* (Carroll, 2011). For the middle childhood group, we used the English language version, and for the adult group, which consisted of native speakers of diverse languages, we used the version in each participant's native language (data from Malik-Moraleda, Ayyash et al., 2022). The passages were recorded by a female native speaker. For the control condition, the stimuli were acoustically degraded, as described originally in Scott et al. (2017). Briefly, the intact files were low-pass filtered at a pass-band frequency of 500 Hz. In addition, a noise track was created from each intact clip by randomizing 0.02-s long periods. To produce variations in the volume of the noise, the noise track was multiplied by the amplitude of the intact clip's signal over time. The noise track was then low-pass filtered at a pass-band frequency of 8,000 Hz and a stop frequency of 10,000 Hz to soften the highest frequencies. The noise track and the low-pass-filtered copies of the intact files were then combined, and the level of noise was adjusted to a point that rendered the clips unintelligible. The resulting degraded clips sound like poor radio reception of speech, in which the linguistic content is not discernible. For all participants in the adult group and a subset of participants in the middle childhood group, a third condition was included, which consisted of passages in an unfamiliar foreign language. Because the acoustically degraded condition i) was presented to all participants, and ii) is the same as the control condition used in Localizer Variant 3 (used for Dataset 1) as well as being more similar to the reverse-speech control condition used in Localizer Variant 1 (used for Dataset 1), we chose to use this condition, instead of the foreign language condition, as the control condition (but see Malik-Moraleda, Ayyash et al., 2022, for evidence that the acoustically degraded and foreign-language conditions both work well as control conditions). Participants were instructed to listen to the clips. Participants who performed the two-condition version completed two runs, each consisting of 6 experimental events per condition and 4 fixation periods (23 s each), for a total run duration of 264 s. Participants who performed the three-condition version completed three runs, each consisting of 4 experimental events per condition and 3 fixation periods (12 s each), for a total run duration of 252 s. In both versions, condition order was palindromic and varied across runs.

Localizer Variant 3 (used for the late childhood group in Dataset 1): The critical condition consisted of short (18 s long) engaging excerpts from diverse sources (e.g., *The Moth* podcast, *TED Talks*, celebrity interviews), as detailed in Scott et al. (2017). For the control condition, the stimuli were acoustically degraded, as described in Scott et al. (2017) and identically to the procedure used for Localizer Variant 2. Participants were instructed to listen to the clips. Participants completed two runs, each consisting of 8 experimental events per condition and 5 fixation periods (14 s each), for a total run duration of 358 s. Condition order was palindromic and varied across runs.

Localizer Variant 4 (used for all participants in Dataset 2): The critical condition consisted of short (20 s long) engaging stories. The stories were recorded by one of three female native English speakers. The stories varied in content and fell into three conditions (Mental—stories about the characters' mental states, Social—stories about the characters' appearance and social relationships, and Physical—stories about physical objects and events in the world); for the purposes of the current study, we combined data from all stories given that in the other localizer variants, the materials in the critical language condition spanned diverse kinds of content, including mental, social, and physical types of events. The control condition consisted of clips in an unfamiliar foreign language. (A fifth condition, of no interest to the current study, included music clips.) The materials used in this Localizer Variant are available on OSF: https://osf.io/cbw6f/. Across conditions, each stimulus was followed by an auditorily presented question ("Does this come next?"; 1.5 s in duration), and then by another short (3 s) probe clip. The clip was followed by a 6.5 s pause during which participants were instructed to respond by pushing one of two buttons ("Yes" or "No"). Correct responses were followed by an encouraging phrase ("Way to go!"), and incorrect response by "Let's try another!". For the language stimuli, half of the stories were followed by the correct ending (and thus required the "Yes" response); incorrect endings were drawn randomly from all other English story conditions. For the control stimuli (unfamiliar foreign language stimuli in Hebrew, Korean, or Russian), half of the stories were followed by a clip in the same language (and thus required the "Yes" response), and the other half by a clip in another language; participants were asked to answer whether the probe clip was in the same language as the previous story. Participants completed four runs, each consisting of 2 experimental events per condition and three rest periods 12 s each), for a total run duration of 6.6 min. A rest block occurred at the start of each run, at the halfway point of each run (after five experimental blocks), and at the end of each run. Condition order was palindromic and varied across runs. (Only the stimuli themselves (20s stories in the participant's native language or unfamiliar foreign language) were modeled as the experimental events.)

Table SI-1. Details of the design, procedure, and timing for the language localizer paradigm variants.

	**Variant 1** (DS1, Early Childhood)	**Variant 2** (DS1, Middle Childhood & Adults)	**Variant 3** (DS1, Late Childhood)	**Variant 4** (DS2, All Age Groups)
Number of conditions	3	3	2	5
Condition names	Critical: Forward; Control: Backward*, Dichotic	Critical: Intact; Control: Degraded*, Foreign	Critical: Intact; Control: Degraded	Critical: Mental, Social, Physical; Control: Foreign*, Music
Critical contrast	Forward > Backward	Intact > Degraded	Intact > Degraded	Average of Mental, Social, Physical > Foreign
Block duration	15 sec	18 sec	18 sec	20 sec (+ task, which was separated in time and modeled separately)
Number of blocks/run	18 (6 per condition)	12 (4 per condition)	16 (8 per condition)	10 (2 per condition)
Total number of blocks for the critical and control conditions	6; 6	12; 12	16; 16	24; 8
Rest block duration	5 sec	12 sec	14 sec	12 sec
Number of rest blocks/ run	18	3	5	3

Run duration	360 sec	252 sec	358 sec	396 sec
Number of runs	1 (divided into 2)	3	2	4

Note: Variant 4 did not use a fixation cross during the rest/fixation period.
* Control condition used for contrast.

SI-2: Magnitude of responses to language in the RH language network across development.

Magnitude of responses to the Language > Control contrast in the RH language network													
		RH language network				RH temporal language areas				RH frontal language areas			
		B	*SE*	*t*	*p*	*B*	*SE*	*t*	*p*	*B*	*SE*	*t*	*p*
DS 1	Early	0.16	0.08	1.95	0.05	0.55	0.11	5.05	**<0.001**	0.1	0.11	0.88	0.378
	Middle	0.82	0.08	9.96	**<0.001**	1.34	0.11	11.85	**<0.001**	0.46	0.09	5.21	**<0.001**
	Late	1.13	0.05	21.14	**<0.001**	1.82	0.08	22.69	**<0.001**	0.67	0.05	12.15	**<0.001**
	Adult	1.06	0.06	17.95	**<0.001**	1.52	0.09	17.68	**<0.001**	0.76	0.07	11.57	**<0.001**
DS 2	Early	0.19	0.12	1.64	0.11	0.41	0.22	1.91	0.07	0.04	0.12	0.37	0.715
	Middle	0.44	0.06	7.45	**<0.001**	0.65	0.08	7.85	**<0.001**	0.3	0.08	3.84	**<0.001**
	Late	0.43	0.07	6.58	**<0.001**	0.67	0.1	6.82	**<0.001**	0.27	0.09	3.21	**0.002**
	Adult	0.35	0.06	5.61	**<0.001**	0.50	0.08	5.98	**<0.001**	0.24	0.08	3.07	**0.003**

Table SI-2. Magnitude of responses to language in the RH language network as a whole, and in the temporal and frontal components separately, across development. For each age group (early childhood, middle childhood, late childhood, and adulthood) in each dataset (DS1, DS2), we fit a linear mixed-effects regression model predicting the magnitude of the BOLD response (estimated in each region as described in Methods-Section7) from condition (Language vs. Control) with random intercepts for participants and fROIs (Methods-Section8). The *p*-values for the temporal and frontal areas are uncorrected, but all significant values survive a Bonferroni correction for two comparisons.

SI-3: Effect of hemisphere on the volume of activation for the Language > Control contrast in the temporal and frontal components of the language network across development.

**Lateralization of the Language > Control activations (Volume)**									
		Temporal language areas (LH > RH)				Frontal language areas (LH > RH)			
		B	*SE*	*t*	*p*	*B*	*SE*	*t*	*p*
DS 1	Early	224.59	74.21	3.03	**0.004**	47.61	16.58	2.87	**0.007**
	Middle	355.96	70.68	5.04	**<0.001**	69.25	24.57	2.82	**0.007**
	Late	819.09	63.86	12.83	**<0.001**	268.81	29.96	8.97	**<0.001**
	Adult	469.03	63.74	7.35	**<0.001**	145.05	22.47	6.46	**<0.001**
DS 2	Early	191.83	171.39	1.12	0.32	93.17	57.62	1.62	0.17
	Middle	163.24	69.73	2.34	0.03	87.34	35.75	2.44	**0.02**
	Late	470.7	145.03	3.25	**0.004**	185.96	53.35	3.49	**0.002**
	Adult	456.69	87.5	5.22	**<0.001**	224.19	42.52	5.27	**<0.001**

Table SI-3. Effect of hemisphere on the volume of activation for the Language > Control contrast in the temporal and frontal components of the language network across development. For each age group in each dataset, we first calculated—in each participant—the total number of significant Language > Control voxels (estimated in each region as described in Methods-Section7). We then fit a linear mixed-effects regression model predicting the total number of voxels from hemisphere (left vs. right) with random intercepts for participants (Methods-Section8). The *p*-values for the temporal and frontal areas are uncorrected, but all significant values survive a Bonferroni correction for two comparisons.

SI-4: Strength of inter-regional correlations among the LH and RH language fROIs.

A. Strength of inter-regional correlations (LH)										
		Language network (correlations among all n=10 region pairs)			Local, within-temporal-lobe (n=1 region pair) and within-frontal-lobe correlations (n=3 region pairs)			Non-local correlations between frontal and temporal regions (n=6 region pairs)		
		r	*t*	*p*	*r*	*t*	*p*	*r*	*t*	*p*
DS1	Early	0.57	19.79	**<0.001**	0.63	21.72	**<0.001**	0.64	22.49	**<0.001**
	Middle	0.60	31.61	**<0.001**	0.66	36.32	**<0.001**	0.66	36.17	**<0.001**
	Late	0.77	103.42	**<0.001**	0.78	103.94	**< 0.001**	0.77	103.42	**<0.001**
	Adult	0.71	60.02	**<0.001**	0.76	65.96	**< 0.001**	0.74	63.01	**<0.001**
**B. Strength of inter-regional correlations (RH)**										
		r	*t*	*p*	*r*	*t*	*p*	*r*	*t*	*p*
DS1	Early	0.57	18.83	**<0.001**	0.73	28.83	**<0.001**	0.43	10.66	**<0.001**
	Middle	0.57	27.01	**<0.001**	0.73	40.15	**<0.001**	0.42	15.08	**<0.001**
	Late	0.69	81.8	**<0.001**	0.81	116.22	**<0.001**	0.59	49.52	**<0.001**
	Adult	0.67	54.67	**<0.001**	0.80	86.89	**<0.001**	0.57	36.55	**<0.001**

Note: Dataset 2 did not include resting state data.

Table SI-4. Strength of inter-regional correlations among the LH (A) and RH (B) language fROIs across development. For each age group in Dataset 1, we first calculated—in each participant—pairwise inter-region correlations within each hemisphere separately (A: LH and B: RH) i) among all fROIs (Language Network), ii) between the two temporal fROIs and among the three frontal fROIs (Local correlations), and iii) for the six frontal-to-temporal fROI pairs (Non-local correlations). We then conducted one-sample t-tests (against zero) on mean z-scored correlation values. The *p*-values are uncorrected, but all significant values survive a Bonferroni correction for three comparisons.

SI-5: Age-related changes in the magnitude of the Language > Control contrast in the LH language network and the strength of inter-regional correlations among LH language fROIs, controlling for motion.

		B	SE	t	*p*
**A. Age differences in the magnitude of the Language > Control contrast, controlling for motion**					
Dataset 1	Age = Continuous (Ages 4-16)	0.12	0.02	5.87	**< 0.001**
	Early vs. Adult	-0.6	0.17	-3.58	**0.002**
	Middle vs. Adult	-0.04	0.15	-0.29	0.99
	Late vs. Adult	0.37	0.14	2.72	**0.03**
	Early vs. Late	-0.97	0.17	-5.63	**<0.001**
	Middle vs. Late	-0.39	0.16	-2.415	**0.04**
	Early vs. Middle	-0.58	0.2	-2.86	**0.01**
Dataset 2	Age = Continuous (Ages 4-16)	0.07	0.03	1.91	0.06
	Early vs. Adult	0.43	0.29	1.47	0.46
	Middle vs. Adult	0.13	0.18	0.72	0.89
	Late vs. Adult	0.163	0.2	0.83	0.84
	Early vs. Late	-0.61	0.28	-2.18	0.08
	Middle vs. Late	-0.37	0.28	-1.13	0.39
	Early vs. Middle	-0.25	0.17	-0.68	0.33
**B. Age differences in the strength of inter-regional correlations, controlling for motion**					
Dataset 1	Age = Continuous (Ages 4-16)	0.8	0.8	63.02	**<0.0001**
	Early vs. Adult	-0.13	0.02	-5.85	**< 0.001**
	Middle vs. Adult	-0.09	0.02	-4.06	**< 0.001**
	Late vs. Adult	0.02	0.02	1.5	0.44
	Early vs. Late	-0.16	0.02	-7.18	**<0.0001**
	Middle vs. Late	-0.11	0.02	-5.44	**<0.0001**
	Early vs. Middle	-0.04	0.03	-1.81	0.17

Table SI-5. Age-related changes in the magnitude of the Language > Control contrast in the LH language network (A), and the strength of inter-regional correlations among LH language fROIs (B). We conducted linear mixed-effects analyses predicting response magnitude and IRC strength of the language network from Age (treated either as a continuous or a categorical variable) with random intercepts for participants and fROIs and, critically, including motion as a covariate. Similar to the main analyses, we then performed pairwise comparisons of the adjusted means for Age using Tukey's method for multiple comparison adjustments. The results are summarized below.

A. After controlling for motion, the analyses where age was treated as a continuous variable revealed a similar pattern as the analyses reported in the main text (without motion as a covariate): a positive effect of age on the magnitude of the Language > Control contrast in Dataset 1 and a positive numerical trend in Dataset 2. In line with these results, in Dataset 1, the Language > Control effect was larger in the late childhood and adult groups compared to the

early childhood groups (*ps*<0.001); the effect was also larger in the late childhood group compared to the middle childhood group ($p<0.05$). In this analysis, the difference between the middle and early childhood groups also came out as reliable ($p<0.05$). None of the other differences came out as significant. In Dataset 2, the results were also similar to what is reported in the main text (without motion as a covariate): a positive but non-significant effect of age; and no significant differences in the pairwise group comparisons.

B. After controlling for motion, the analyses where age was treated as a continuous variable revealed a similar pattern as the analyses reported in the main text (without motion as a covariate): a positive effect of age on the strength of inter-regional correlations. In line with these results, the correlations were stronger in the late childhood and adult groups compared to the early and middle childhood groups (*ps*<0.001); the early and middle childhood groups did not differ significantly, and the late childhood group did not differ from adults. It is also worth noting that age was not a significant predictor of motion ($F(2,188)=3.27$, $p=0.07$).

SI-6: Group-constrained Subject-Specific (GSS) analysis of the pediatric participants.

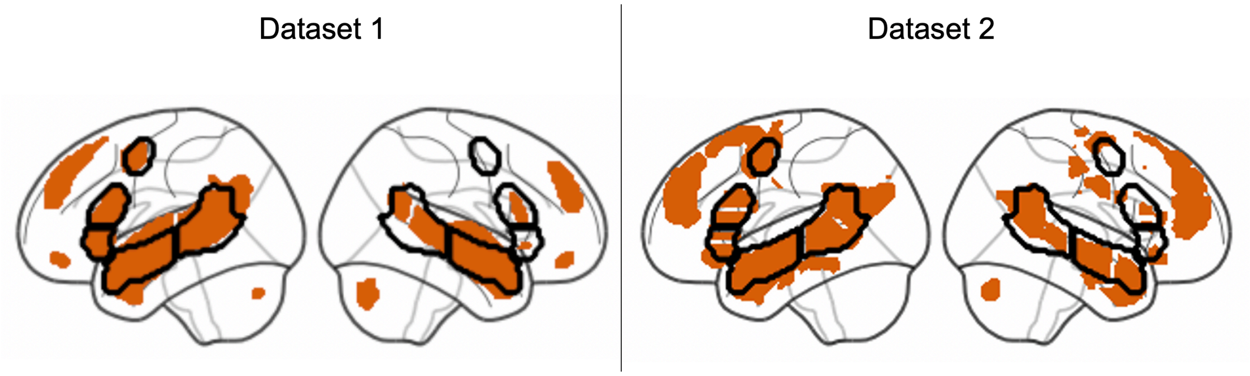

Figure SI-6. Group-constrained Subject-Specific (GSS) analysis of the pediatric participants. In all analyses in the current paper, we used language parcels (masks) that were derived from adult data. This approach makes comparisons with adults straightforward, but also makes an assumption that the general topography of the language activations is consistent across development. To test whether this assumption is reasonable, we performed a GSS analysis (Fedorenko et al., 2010; Julian et al., 2012) on the current pediatric datasets to see if the parcels that are derived from pediatric data are similar to those derived from adults. To perform this analysis, we followed the procedure outlined in Lipkin et al. (2022): in particular, we i) selected the top 10% of voxels across the brain for the Language > Control contrast from each individual participant, ii) binarized the resulting maps so that voxels in the top 10% set get assigned a value of 1 and the rest of the voxels – 0, iii) overlaid the binarized maps in the MNI space to derive a probabilistic overlap map, and finally, iv) parcellated the overlap map using a watershed algorithm. The parcels that resulted for each pediatric dataset are shown in orange, and the adult parcels are overlaid in black outlines. As can be seen, the adult parcels overlap near-perfectly with the parcels derived from the pediatric data, at least in the left hemisphere (in the right hemisphere, the pediatric parcels look more noisy, in line with weaker responses in the right hemisphere and overall weaker responses in younger participants). It is worth noting that the parcels in the pediatric data that are not included in the adult set (e.g., the medial frontal parcels) do exist in the adult data too (see e.g., Fedorenko et al., 2010; Lipkin et al., 2022), but are excluded from the adult set due to the current paper's focus on the 'core' lateral frontal and lateral temporal areas.

SI-7. Within- and between-participant correlations of the fine-grained activation patterns for the Language > Control contrast in Dataset 1.

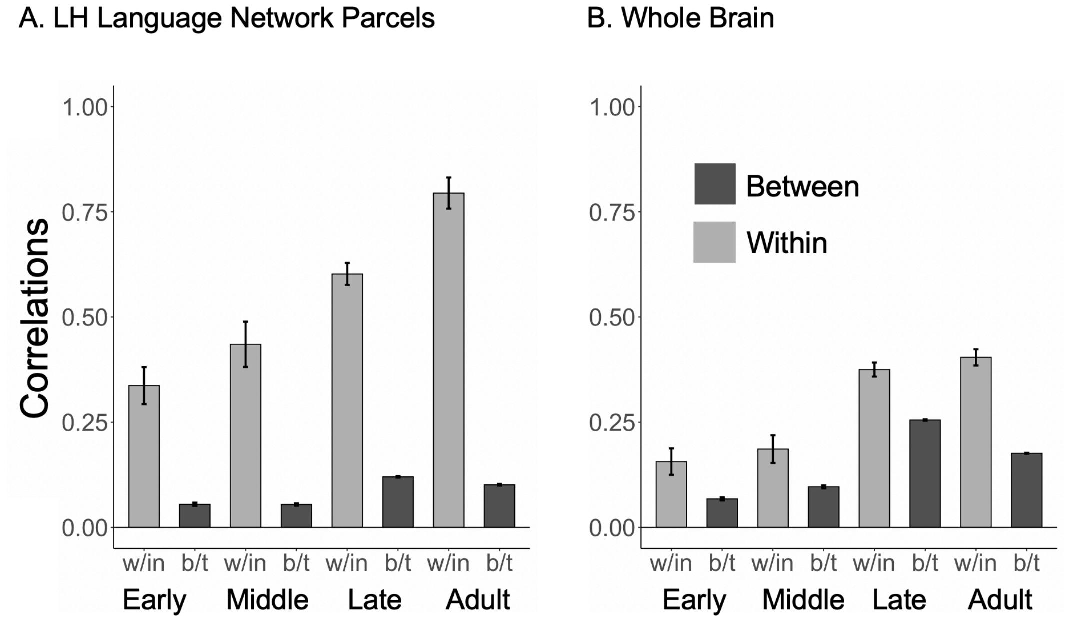

Figure SI-7. Within- and between-participant correlations of the fine-grained activation patterns for the Language > Control contrast in Dataset 1 for the language parcels (A) and across the brain (B). This analysis was performed to evaluate the stability of individual-participant activation maps for the Language > Control contrast across development, and the degree of inter-individual variability. Within-participant correlations are shown in lighter grey, between-participant correlations are shown in dark grey.

A. Within-participant (across-run) and between-participant correlations of the activation patterns for the Language > Control contrast within the LH language parcels. The between-participant correlations were computed by correlating the activations of each participant with that of every other participant in that age group, and then averaging the values to derive a single value per participant.

B. Same as in A, but not restricting the voxels to fall within the LH language parcels.

As quantified by the statistical analyses below, the stability of the activation patterns (both within the LH language parcels and across the brain) increases with age, mirroring the effects observed for the magnitude of responses to the Language > Control contrast (Results section 3). The correlations are in the moderate to high range within the language parcels (r=0.34 to 0.79) and low to moderate range across the brain (r=0.16 to 0.40). The between-participant correlations also show a small increase with age, but critically, the between-participant correlations are consistently much lower than the within-participant correlations, even in young children (within language parcels: r=0.05 to 0.1, across the brain: r=0.07 to 0.25), which provides a strong motivation for using individual-level analytic approaches that take into account the variability in the precise locations of the language areas (see Fedorenko et al., 2010, for discussion).

**A. Age differences in within-participant correlations (indexing activation stability) within the language network.**				
	B	***SE***	**t**	***p***
Age = Continuous	0.04	0.01	5.80	**<0.001**
Early vs. Adult	-0.46	0.06	-7.22	**<0.001**
Middle vs. Adult	-0.36	0.06	-6.54	**<0.001**
Late vs. Adult	-0.19	0.05	-4.00	**0.001**
Early vs. Late	-0.27	0.06	-4.30	**<0.001**
Middle vs. Late	0.17	0.05	3.15	**0.01**
Early vs. Middle	-0.10	0.07	-1.46	0.464
**B. Age differences in within-participant correlations (indexing activation stability) across the brain.**				
	B	***SE***	**t**	***p***
Age = Continuous	0.03	0.00	6.69	**<0.001**
Early vs. Adult	-0.25	0.04	-6.70	**<0.001**
Middle vs. Adult	-0.22	0.03	-6.56	**<0.001**
Late vs. Adult	-0.03	0.03	-1.04	0.881
Early vs. Late	-0.22	0.04	-6.06	**<0.001**
Middle vs. Late	-0.19	0.03	-5.87	**<0.001**
Early vs. Middle	-0.03	0.04	-0.74	0.727
**C. Age differences in between-participant correlations (indexing inter-individual topographic variability) within the language network.**				
	B	***SE***	**t statistic**	***p***
Age = Continuous	-0.01	0.00	-10.39	**<0.001**
Early vs. Adult	-0.05	0.01	-6.31	**<0.001**
Middle vs. Adult	-0.05	0.01	-7.04	**<0.001**
Late vs. Adult	-0.02	0.01	-3.42	**0.004**
Early vs. Late	-0.07	0.01	-9.12	**<0.001**
Middle vs. Late	0.07	0.01	10.23	**<0.001**
Early vs. Middle	0.00	0.01	0.01	1.00
**D. Age differences in between-participant correlations (indexing inter-individual topographic variability) across the brain**				
	B	***SE***	**t**	***p***
Age = Continuous	0.03	0.00	15.30	**<0.001**
Early vs. Adult	-0.11	0.12	-9.15	**<0.001**
Middle vs. Adult	-0.08	0.01	-7.56	**<0.001**
Late vs. Adult	0.08	0.01	8.45	**<0.001**
Early vs. Late	-0.01	0.01	-15.80	**<0.001**
Middle vs. Late	0.16	0.01	14.99	**<0.001**
Early vs. Middle	-0.03	0.01	-2.17	0.134

Table SI-7. Age differences in the within- and between-subject correlations across the whole brain in Dataset 1. For all participants across the three childhood groups, we fit a linear regression model predicting the correlation coefficients from age as a continuous variable. We then re-ran the linear regression model using age group as a categorical predictor, followed by inter-group pairwise comparisons adjusted using Tukey's method.

SI-8. Correlations between the Language > Control magnitude in the LH language network and the strength of inter-regional correlations among the LH language fROIs in Dataset 1.

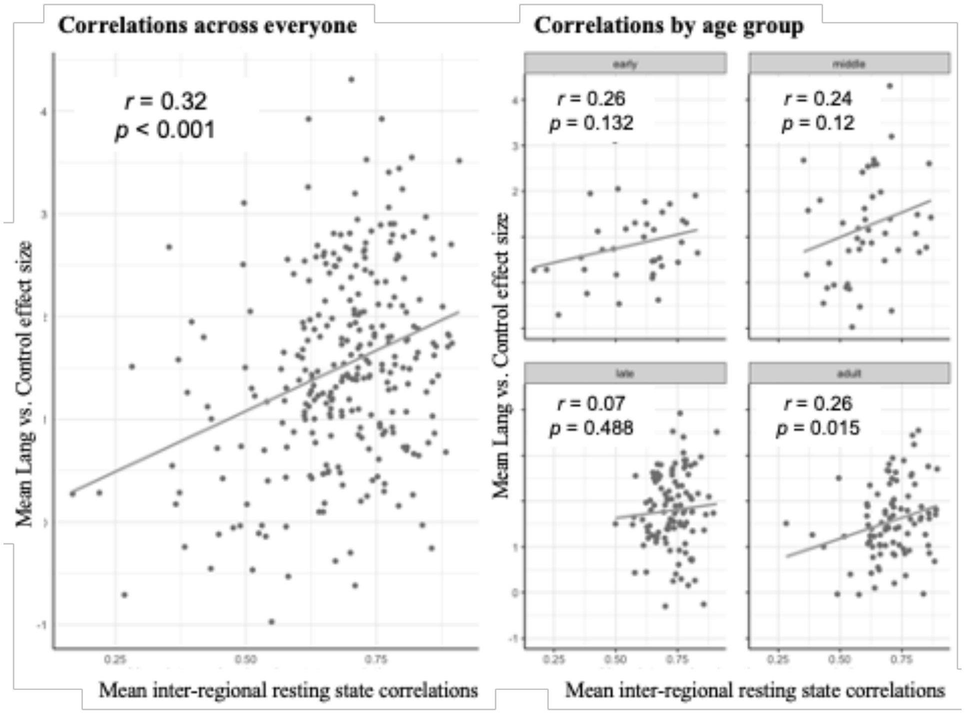

Figure SI-8. Correlations between the Language > Control magnitude in the language network and the strength of inter-regional correlations among the LH language fROIs in Dataset 1. Although these two neural markers of the language network activity show a positive relationship across all participants and in three of the four age groups, the correlations are only moderate, which suggests that they may reflect at least partially dissociable aspects of language development.

Figure SI-9. A systematic comparison between the data from the current study and the data and analyses from Olulade et al.'s (2020) study with respect to the lateralization of the language network.

Summary of Olulade et al.'s design and the critical lateralization analysis.

Olulade et al. (2020) examined neural responses during a language task in a group of n=53 participants across four age groups (n=39 children and 14 adults; the age range for the children was similar to the range in the current study). Each participant performed a single run of a 2-condition experiment. In the *critical* (forward speech) condition, participants listened to short sentences and were asked to press a button at the end to decide whether the sentence was factually correct. In the *control* (backward speech) condition, participants listened to the same sentences played backwards and were asked to press a button at the end to decide whether a beep was present at the end of the sound file. No fixation condition was included. The key claims in Olulade et al. are that i) the language system is more bilateral in children and becomes increasingly left-lateralized with age, and ii) the change in the degree of lateralization is due to a decrease in the activity of the RH language areas with age. The following analysis was performed by Olulade and colleagues to evaluate these claims:

- Individual-level activation maps were thresholded at $p<0.001$ uncorrected at the whole-brain level and evaluated against two anatomical right-hemisphere masks (ROIs). One covered the inferior frontal gyrus, and the other covered lateral temporal and parietal cortex.
- For each age group and for each ROI separately: if a participant had a cluster of size=269 voxels or larger, they were assigned a value of 1, and otherwise they were assigned a value of 0.
- In each group and for each ROI, the values were summed, divided by the number of participants in that group, and multiplied by 100% to convert from proportions to percentages.

By this measure, Olulade et al. found that in the youngest age group, almost every participant showed language activation in the RH ROIs, but in the older groups the proportion of participants with RH activation decreased. These results appear in Figure 4 in Olulade et al.'s paper (repeated in the top row in **Figure SI-9A** below).

This result appears inconsistent with the results we report for our data, where we do not see a change in language lateralization as a function of age in either dataset: even the youngest children in our datasets show left-lateralized responses, and the strength of lateralization is similar to that observed in the adult group. However, the comparison with Olulade et al.'s results is challenging given that the paradigms, the preprocessing and modeling pipelines, and the measure used to assess lateralization differ between their study and ours. To facilitate the comparison, we performed two analyses, as described below. First, we show that Olulade et al.'s results are reproducible by implementing their preprocessing and modeling pipeline and using their measure of lateralization. And second, we show that when using our pipeline and more standard measures of lateralization, the results are not consistent with Olulade et al.'s claims and

instead align with what we find in the current study. We then elaborate on the likely source of the discrepancy.

Analysis 1: Evaluating the *reproducibility* of Olulade et al.'s (2020) findings.

Data: Olulade et al.'s data
Preprocessing + analysis: Olulade et al.'s pipeline with one change in how outlier timepoints were modeled
Lateralization measure: Olulade et al.'s measure (proportion of participants with significant activation in RH frontal and temporal masks)

We ran the data from Olulade et al.'s study through a preprocessing and modeling pipeline which was designed to be as similar as possible to the one used in their study. We thank Elissa Newport and Anna Greenwald for providing us with 3D nifti files, realignment parameter (rp) files, bad scan regressor files, segmentation images for WM and GM, and ROI masks. The only change we made to the modeling pipeline was to use a GLM regressor for *each* outlier scan rather than a single regressor for all outlier scans as in Olulade et al.

Motivation for the change in how the outliers are modeled: Using a single regressor for all outlier scans is problematic. If a) the number of outliers varies between conditions (what is commonly referred to as "task-related motion"), and b) outliers are not modeled correctly, the residual/uncorrected outlier effects can show up as artifactual task effects (inflating both the effect sizes and the associated statistics). Because motion effects are rather large compared to typical task effects, they can easily dominate the results. Indeed, in Olulade et al.'s data, participants (and especially younger kids) moved much more during the control (reverse speech) condition compared to the critical (forward speech) condition. As a result, we wanted to ensure that their results held when the outliers were modeled correctly.

To additionally evaluate the robustness of the results to the choice of the number of voxels above which a participant is considered to have RH activation, we report the results for four thresholds: at least 1 significant voxel, at least 10 significant voxels, at least 100 significant voxels, and at least 200 significant voxels.

We find that the results reported in Olulade et al. (included here in the top row in **Figure SI-9A** below, for ease of comparison) are reproducible when the outliers are modeled by including a regressor for each outlier scan and are robust to the choice of the threshold, showing the same downward trend in the proportion of participants with significant RH activation with age in both the frontal and temporo-parietal ROIs. However, the effects are substantially reduced in size: for example, for the frontal ROI, the difference reported in Olulade et al. between the youngest group and adults is ~75%, but in our re-analysis, the differences are on the order of 20-60% across thresholds, which suggests that improper modeling of motion outliers may have contributed to the results reported in Olulade al.'s paper.

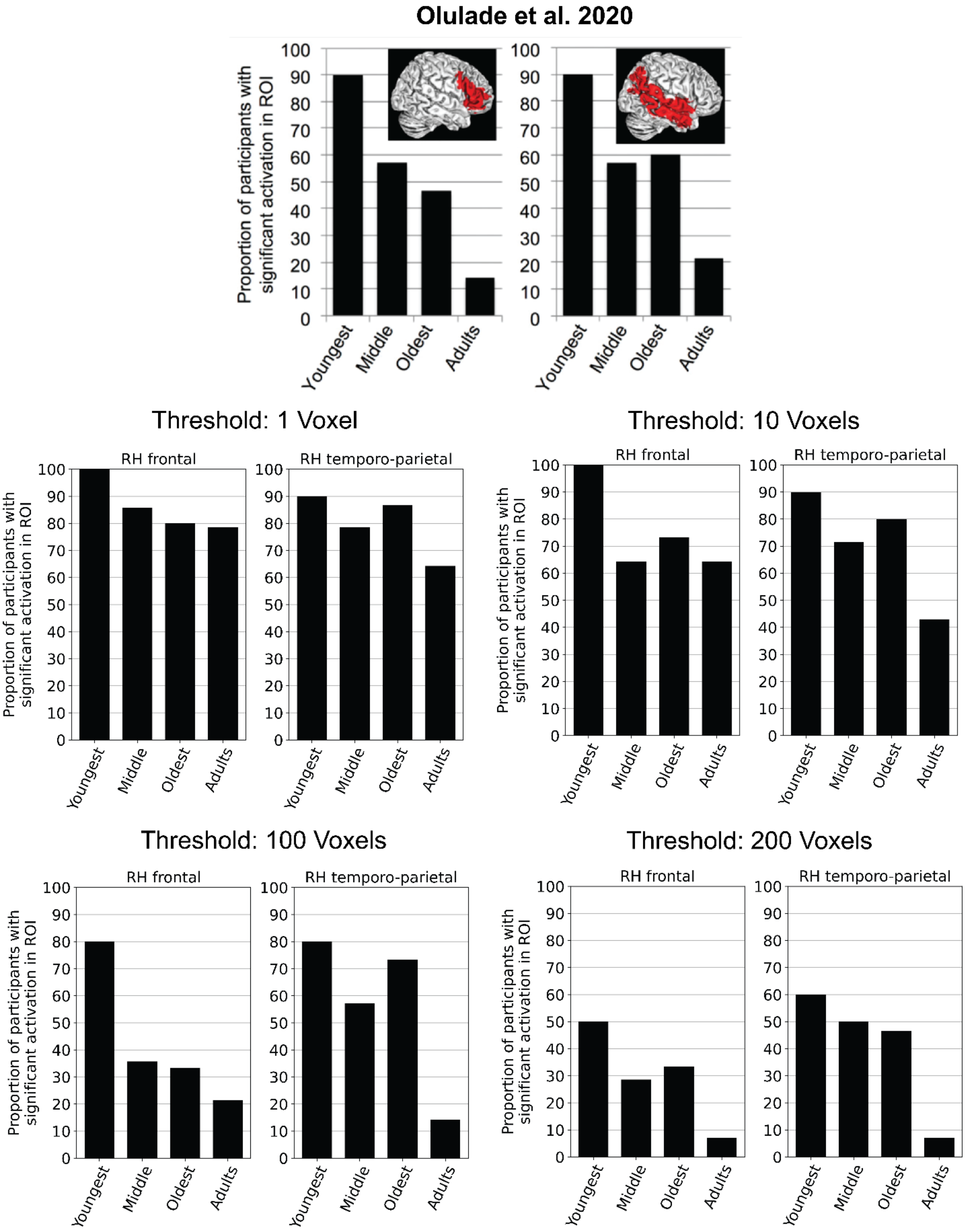

Figure SI-9A: A comparison between Olulade et al.'s (2020) results as reported in the paper vs. a re-analysis of their data. The data are re-analyzed in a slightly modified modeling pipeline (as

discussed above) and the lateralization effects are computed across several thresholds of how many voxels are needed to count the participant as having RH activation (1 voxel, 10 voxels, 100 voxels, and 200 voxels). The pattern reported by Olulade et al. is reproducible and robust to the choice of threshold, although the effects are reduced in size.

Analysis 2: Evaluating language lateralization in Olulade et al.'s (2020) and our data when using the current study's preprocessing and modeling pipeline and examining more standard measures of lateralization.

Data: Olulade et al.'s and our data
Preprocessing + analysis: The pipeline used in the current study
Lateralization measure: Measures used in the current study (volume-based lateralization index and the number of LH vs. RH language-responsive voxels within the language masks; see Methods-Section7)

We ran the data from Olulade et al.'s study through the preprocessing and modeling pipeline that was used in the current study. The key differences between the two pipelines are summarized in Table SI-9A.

	Olulade et al. 2020 pipeline	***Current pipeline***
Outlier regressors	Single regressor for all outlier scans	Single regressor per outlier scan
Smoothing of functional data	8mm FWHM kernel	4mm FWHM kernel
Temporal response function	Canonical hemodynamic response function (HRF)	Canonical hemodynamic response function (HRF) and first-order temporal derivative
Global scan regressor	Included	Not included
Task design blocks	The first experimental block of the run is specified as starting at the 2nd instead of the 1st TR (1 TR = 3 sec difference).	The first experimental block of the run is specified as starting at the 1st TR.

Table SI-9A. Key differences between Olulade et al.'s (2020) vs. our preprocessing and modeling pipelines.

Before diving into the results, it is worth noting that Olulade et al. motivate their measures as follows (bolding is ours to highlight the critical bits of the argument):

> *"The vast majority of studies to date have investigated language lateralization using a laterality index (LI) comparing LH and RH activation. While the precise method for quantifying LH and RH activation and computing the LI differs across studies (40, 41), the LI in general compares the difference between LH and RH activation with the total activation. LIs near 0 indicate bilateral and equal activation in the two hemispheres, whereas positive*

LIs indicate left lateralization. This measure allows quantification of lateralization regardless of absolute activation levels, which may be influenced by age or other factors that may differ over age, such as stimulus complexity, effort, and task difficulty (17, 42–45). However, because the LI is a difference score, ***potentially important information may be lost****. An increase in LI with age could reflect a decrease of RH activation, an increase of LH activation, or both; the extent to which the LH and the RH are each involved in language processing is not directly reflected in LI scores. […] For these reasons, the present fMRI study focuses on* ***activation patterns in each hemisphere*** *rather than on lateralization per se […].”*

However, Olulade et al. do not report the amount of LH and RH activation (in magnitude or spatial extent) separately; they only report the *proportion of participants with RH activation* (above some fixed number of voxels; see Analysis 1 above). Below, we report LI measures as well as volumes of LH and RH activation.

The voxel count data (bottom row in **Figure SI-9B**) reveal that Olulade et al.'s data are not consistent with the key empirical claim in their paper (i.e., that RH activation in the language areas decreases with age). In particular, in their dataset, the number of language-responsive voxels in the RH remains relatively constant across the four age groups (similar to our Dataset 2), but the number of language-responsive voxels in the LH increases. This pattern leads to a slight age-related increase in the lateralization index scores, from ~0.4 in the early childhood group to ~0.6 in adults (top row in **Figure SI-9B**). Note, however, that all four groups—including the youngest children—already show *strongly left-lateralized language activations* (all LI measures are positive, and the number of LH language-responsive voxels is larger than the number of RH language-responsive voxels), similar to what we observe in both of our datasets (second and third columns in **Figure SI-9B**).

So, what is the **possible source of the discrepancy** between Olulade et al.'s reported results (reproduced here qualitatively in Analysis 1 above) and our re-analysis of their data? As briefly mentioned in the main text, the difference plausibly has to do with i) Olulade et al.'s reliance on a paradigm that conflates linguistic and general cognitive processing, and ii) their use of masks for voxel-count analyses that encompass both language and domain-general areas. Let us unpack these points.

First, our study used an extensively validated language localizer paradigm (Fedorenko et al., 2010). This paradigm robustly differentiates the language network from the nearby domain-general Multiple Demand (MD) network (Duncan, 2010, 2013)—a bilateral network of frontal, inferior temporal, and parietal areas whose different components lie adjacent to the frontal and temporal language areas (Fedorenko et al., 2012; Fedorenko & Blank, 2020; Braga et al., 2020; DiNicola et al., 2024). The MD network is strongly sensitive to task demands across domains (e.g., Duncan & Owen, 2000; Fedorenko et al., 2013; Hugdahl et al., 2015; Shashidhara et al., 2020), and language tasks where linguistic processing is accompanied by general task demands engage the MD network in addition to the language network (Diachek, Blank, Siegelman et al., 2020).

The language task used in Olulade et al. study conflates linguistic and general task demands: the critical condition requires linguistic processing but it is also *more cognitively demanding* than the

control condition. As a result, this contrast will engage both the language network and the Multiple Demand network. Moreover, task demands vary across the age groups (see the fourth row in Table 1 in Olulade et al., which shows a gradual increase in the accuracy of responses across the four age groups; although the accuracies are not broken down by condition, the differences most likely stem from the critical condition given that the task in the control condition is very easy: to press a button if a beep is heard). Given these age-related differences in task difficulty, the reliance on the MD network likely also varies across age groups. In particular, younger children plausibly engage the Multiple Demand network to a greater extent than older children and adults, which would result in overall more bilateral activations given that the MD network is bilateral. As the task becomes easier with age, the reliance on the MD network is likely reduced, and the language network becomes dominant, which results in more left-lateralized activations (given that the language network is typically left-lateralized).

And **second**, Olulade et al. used large anatomical masks as their ROI masks. These masks encompass parts of both the language network and the MD network (see e.g., Fedorenko & Blank, 2020; Braga et al, 2020). Given the use of a paradigm that likely engages both networks, these ROI masks capture effects that arise in both of these networks, making it impossible to unambiguously attribute the effects to one or the other network.

Future studies should either use the validated localizers that have already been established to isolate the language-processing mechanisms from those that support cognitive demands associated with task performance, or—if a novel task is used that potentially conflates linguistic and general cognitive processing—they should design the paradigm in a way such that the task component can be modeled separately from the language-processing component of the task (as in Dataset 2 in the current study), and/or include a localizer for the MD network to help rule out the contribution of this network if the research questions focus on the language network.

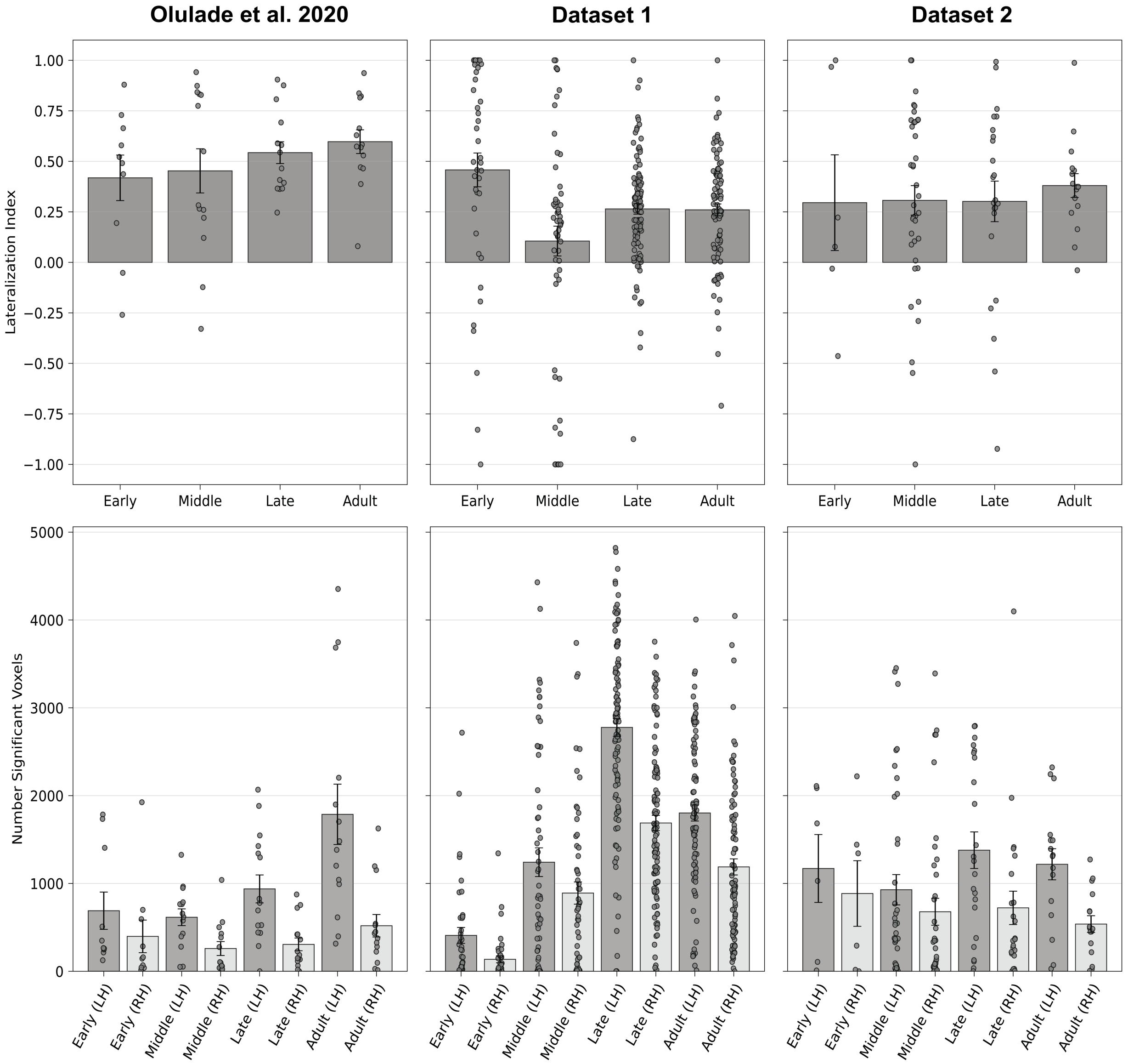

Figure SI-9B: A comparison between Olulade et al.'s (2020) data (left column) and our data from Datasets 1 (middle column) and 2 (right column) when all data are analyzed through the same preprocessing and modeling pipeline (used in the current study) and the lateralization effects are examined with standard measures. The top row shows volume-based lateralization index measures and the bottom row shows activation volumes broken down by hemisphere.

SI References

Bedny, M., Pascual-Leone, A., Dodell-Feder, D., Fedorenko, E., & Saxe, R. (2011). Language processing in the occipital cortex of congenitally blind adults. *Proceedings of the National Academy of Sciences of the United States of America*, *108*(11), 4429–4434. https://doi.org/10.1073/pnas.1014818108

Carroll, L. (2011). *Alice's Adventures in Wonderland*. Broadview Press.

Duncan, J., & Owen, A. M. (2000). Common regions of the human frontal lobe recruited by diverse cognitive demands. *Trends in neurosciences*, *23*(10), 475–483. https://doi.org/10.1016/s0166-2236(00)01633-7

Fedorenko, E., Hsieh, P.-J., Nieto-Castañón, A., Whitfield-Gabrieli, S., & Kanwisher, N. (2010). New method for fMRI investigations of language: Defining ROIs functionally in individual subjects. *Journal of Neurophysiology*, *104*(2), 1177–1194. https://doi.org/10.1152/jn.00032.2010

Fedorenko, E., Duncan, J., & Kanwisher, N. (2013). Broad domain generality in focal regions of frontal and parietal cortex. *PNAS Proceedings of the National Academy of Sciences of the United States of America, 110*(41), 16616–16621. https://doi.org/10.1073/pnas.1315235110

Hugdahl, K., Raichle, M. E., Mitra, A., & Specht, K. (2015). On the existence of a generalized non-specific task-dependent network. Frontiers in human neuroscience, 9, 430. https://doi.org/10.3389/fnhum.2015.00430

Julian, J. B., Fedorenko, E., Webster, J., & Kanwisher, N. (2012). An algorithmic method for functionally defining regions of interest in the ventral visual pathway. *NeuroImage, 60*(4), 2357 2364. https://doi.org/10.1016/j.neuroimage.2012.02.055

Malik-Moraleda, S., Ayyash, D., Gallée, J., Affourtit, J., Hoffmann, M., Mineroff, Z., ... & Fedorenko, E. (2022). An investigation across 45 languages and 12 language families reveals a universal language network. Nature Neuroscience, 25(8), 1014-1019. https://doi.org/10.1038/s41593-022-01114-5

Olulade, O. A., Seydell-Greenwald, A., Chambers, C. E., Turkeltaub, P. E., Dromerick, A. W., Berl, M. M., Gaillard, W. D., & Newport, E. L. (2020). The neural basis of language development: Changes in lateralization over age. *Proceedings of the National Academy of Sciences of the United States of America*, *117*(38), 23477–23483. https://doi.org/10.1073/pnas.1905590117

Petersen, D.B., & Spencer, T.D. (2012). The Narrative Language Measures: Tools for Language Screening, Progress Monitoring, and Intervention Planning. Perspectives on Language Learning and Education, 19, 119-129. https://doi.org/10.1044/lle19.4.119

Scott, T. L., Gallée, J., & Fedorenko, E. (2017). A new fun and robust version of an fMRI localizer for the frontotemporal language system. Cognitive neuroscience, 8(3), 167-176. https://doi.org/10.1080/17588928.2016.1201466

Shashidhara, S., Spronkers, F. S., & Erez, Y. (2020). Individual-subject functional localization increases univariate activation but not multivariate pattern discriminability in the "multiple-demand" frontoparietal network. Journal of cognitive neuroscience, 32(7), 1348-1368. https://doi.org/10.1162/jocn_a_01554
